# Related Endogenous Retrovirus-K Elements Harbor Distinct Protease Active Site Motifs

**DOI:** 10.3389/fmicb.2018.01577

**Published:** 2018-07-18

**Authors:** Matthew G. Turnbull, Renée N. Douville

**Affiliations:** ^1^Department of Biology, University of Winnipeg, Winnipeg, MB, Canada; ^2^Department of Immunology, University of Manitoba, Winnipeg, MB, Canada

**Keywords:** endogenous retrovirus-K (ERVK), protease, protease inhibitor, active site motifs, RNAseq, amyotrophic lateral sclerosis, breast cancer, prostate cancer

## Abstract

**Background:** Endogenous retrovirus-K is a group of related genomic elements descending from retroviral infections in human ancestors. HML2 is the clade of these viruses which contains the most intact provirus copies. These elements can be transcribed and translated in healthy and diseased tissues, and some of them produce active retroviral enzymes, such as protease. Retroviral gene products, including protease, contribute to illness in exogenous retroviral infections. There are ongoing efforts to test anti-retroviral regimens against endogenous retroviruses. Herein, we examine the potential activity and diversity of human endogenous retrovirus-K proteases, and their potential for impact on immunity and human disease.

**Results:** Sequences similar to the endogenous retrovirus-K HML2 protease and reverse transcriptase were identified in the human genome, classified by phylogenetic inference and compared to Repbase reference sequences. The topologies of trees inferred from protease and reverse transcriptase sequences were similar and agreed with the classification using reference sequences. Surprisingly, only 62/480 protease sequences identified by BLAST were classified as HML2; the remainder were classified as other HML groups, with the majority (216) classified as HML3. Variation in functionally significant protease motifs was explored, and two major active site variants were identified – the DTGAD variant is common in all groups, but the DTGVD motif appears limited to HML3, HML5, and HML6. Furthermore, distinct RNA expression patterns of protease variants are seen in disease states, such as amyotrophic lateral sclerosis, breast cancer, and prostate cancer.

**Conclusion:** Transcribed ERVK proteases exhibit a diversity which could impact immunity and inhibitor-based treatments, and these facets should be considered when designing therapeutic regimens.

## Introduction

Retroviridae is a diverse family composed of both exogenous infectious viral species whose life cycle includes stages with a ssRNA genome inside virions which is converted into a dsDNA provirus, and endogenous proviral species transmitted in a Mendelian fashion. The evolutionary pressures on ERVs and exogenous retroviruses (XRVs) are very different; ERVs experience much stronger negative selection against pathogenicity since their survival depends directly on reproduction of their host (Holmes, 2011; Stewart et al., 2011; Barbeau and Mesnard, 2015). If fact, some ERVs are directly involved in placentation (Mi et al., 2000), and some host genes descend from retroviral ancestors (Kaneko-Ishino and Ishino, 2012). Indeed, the role of ERVs in the evolution and function of their host genome is becoming more clear (Bannert and Kurth, 2004; Cowley and Oakey, 2013). The apparent inactivity of many ERVs is probably a direct consequence of this pressure, as many ERV integrations contain inactivating mutations that disrupt the function or expression of viral genes. The most severe inactivating mutation is the case of solo LTRs, where the entire coding region of the ERV is deleted. Despite this, a surprising number of ERVs appear to lack obvious inactivating mutations, and some are even infectious, blurring the line between ERV and XRV (Boller et al., 2008; Jha et al., 2011; Denner and Young, 2013; Kozak, 2014). Although no infectious ERV has been proven to exist in the human genome, some loci are polymorphic and population genetics analysis and functional studies have not ruled out low-level ongoing infectious replication of some human ERVs (Turner et al., 2001; Jha et al., 2011; Naveira et al., 2014; Wildschutte et al., 2014, 2016).

Here we focus on the *Betaretrovirus*-like human ERVK, and particularly the HML2 clade (HK2), which includes loci encoding functional enzymes, and whose youngest members may be less than 200,000 years old (Franklin et al., 1988; Boller et al., 2008). Actively transcribed ERVs, such as *ERVK-10*, are assigned names by the Human Gene Nomenclature Committee as recommended by Mayer et al. (2011). The biological role of transcribed ERVK elements is becoming more clear in recent years (Ruda et al., 2004; Macfarlan et al., 2012; Manghera et al., 2014, 2015; Michaud et al., 2014; Schlesinger et al., 2014; Grow et al., 2015; Li et al., 2015; Bray et al., 2016; Christensen, 2016; Becker et al., 2017; Prudencio et al., 2017), but the effects of individual viral proteins is understudied as compared to their XRV counterparts. Protease-mediated maturation of retroviral structural proteins and enzymes is critical to the XRV life-cycle; viruses lacking PR activity produce virions with an immature morphology and are not infectious (Kohl et al., 1988). Even though ERVs are not known to replicate by infection of new cells, they can increase in copy number by reinfection of their host cell; this process called reintegration requires the activity of all the viral enzymes (Dewannieux et al., 2006). Because PR function is conserved across *Retroviridae*, a virus with an inactive protease may be complemented by another retroviral protease. The factors which permit or exclude this complementation must be context dependent; for example, the HK2 protease encoded by *ERVK-10* cleaves HIV-1 Gag *in vitro*, although not *in virio* (Towler et al., 1998). The contextual nature of complementation is not perfectly understood, so care must be taken in applying findings across model systems, and particularly in the context of human disease (Towler et al., 1998; Contreras-Galindo et al., 2012; Morandi et al., 2015).

Structural analysis proceeds most easily using crystallographic structural determination, which is not yet available for the HK2 PR; however, biochemical and genetic analyses reveal a typical A2 aspartic protease, with each 106-residue monomer of the mature dimer processed autocatalytically from a larger precursor (Mueller-Lantzsch et al., 1993; Towler et al., 1998; Kuhelj et al., 2001; Dunn et al., 2002). The active HK2 PR is moderately sensitive to pepstatin, with a pH optimum around 4.5 (Towler et al., 1998; Kuhelj et al., 2001). Some HK2 elements (such as *ERVK-10*) encode a functional protease, and HK2 virions with condensed cores have been observed budding from human cells (Mueller-Lantzsch et al., 1993; Sauter et al., 1995; Towler et al., 1998; Boller et al., 2008). Furthermore, the two reconstructed HK2 viruses *Phoenix* and ERVK_CON_ have a PR-dependent infectivity (Dewannieux et al., 2006; Lee and Bieniasz, 2007). Although the substrate affinity of the HK2 PR has not been extensively studied, the PR cleavage sites in the HK2 Gag polyprotein are known (Kraus et al., 2011), as is its susceptibility to select PIs (Towler et al., 1998; Kuhelj et al., 2001).

In addition to catalyzing maturation of viral proteins, PR may play a role in immunity and pathology of human diseases. Protease pathogenicity has been best studied in HIV-1, whose PR is known to cleave a variety of host proteins (Shoeman et al., 1991; Impens et al., 2012), and to trigger apoptosis under specific conditions (Shoeman et al., 1991; Rumlova et al., 2014). HIV-1 PR also directly combats innate immunity by trafficking the dsRNA sensor RIG-I to the lysosome (Solis et al., 2011). Another example of HIV PR inhibition of innate immunity is its ability to cleave RIPK1 and RIPK2 proteins, thus successfully abrogating NF-κB signaling (Wagner et al., 2015). The effect of proteases is not limited to innate immune signaling proteins, but also effector proteins such as intrinsic restriction factors. Both FIV and MLV cleave APOBEC3 in their respective hosts as a means to evade this antiviral defense (Abudu et al., 2006; Yoshikawa et al., 2017). Given the known pathogenic potential of retroviral proteases, it seems prudent to explore the potential effects of PRs encoded by ERVK which, in contrast to HIV-1, are present in all human beings – and which have been associated with specific human diseases, such as ALS, schizophrenia, rheumatic disease and cancer (Frank et al., 2005; Seifarth et al., 2005; Reynier et al., 2009; Douville et al., 2011; Li et al., 2015; Christensen, 2016).

Here, we undertake genomic, sequence-function, and transcriptomic analysis of the diverse ERV PRs in the human genome. We draw on the existing ERV and XRV literature to evaluate the phylogenetic distribution of sequence motifs with potential functional relevance, focusing on HK2 PRs. We accomplish this by identifying human ERV protease sequences, inferring their phylogenetic affiliation to each other, and comparing them to representative sequences. The classification process was carried out in parallel using RT as a standard comparator. Here we show that distinct ERVK PR variants with predicted differences in their functional activity are differentially transcribed in disease-relevant human tissues.

## Results

### Representative Retroviral Motifs

With the goal of using the most recent human genome build GRCh38 to identify loci encoding retroviral PR and RT enzymes, HMMs were used as a search tool. Pfam-A was searched for all relevant protein families related to retroviruses. **Table 1** describes the output from this search, revealing 92 HMMs in total, with 40 directly related to retroviral core and accessory proteins. Most important amongst these are the retroviral protease domain RVP, and the RT core domain RVT_1. Retrovirus-associated HMMs (**Table 1**), the PR sequence of *ERVK-10* (Towler et al., 1998), and the RVT_1 domain of *Phoenix* (Dewannieux et al., 2006), were subsequently used to identify *pro* and *pol* sequences in representative retroviral sequences and in the human genome.

**Table 1:** Retroviral HMMs from Pfam sorted according to the retroviral protein product from which they were derived.

Protein	Pfam HMMs		
• MA	• Gag_p10	• Gag_p12	• zf-CCHC_5
• CA	• zf-CCHC	• Gag_p30	• Gag_p17
• NC	• Gag_p24	• zf-H2C2	• Gag_p19
	• Gag_MA	• gag_pre-integrs	• Retro_M
	• Retrotrans_gag		
• PR	• Asp_protease_2	• RVP	• Spuma_A9PTase
	• gag-asp_protease	• RVP_2	
• RT	• RVT_1	• RVT_3	• RVT_N
	• RVT_2	• RVT_connect	• RVT_thumb
• RH	• RNase_H		
• IN	• IN_DBD_C	• Rve	• Integrase_Zn
• SU	• GP41	• HERV-K_env_2	• Env_gp36
	• Avian_gp85	• MMTV_SAg	
• DU	• dUTPase		
• Accessory proteins	• Deltaretro_Tax	• Myc-N	• Tax
	• F-protein	• Myc-LZ	• VPR
• Other	• SPX	• Ribosomal_L22	• UBN2_2
	• Borrelia_orfX	• Chromo	• UBN2_3
	• Gypsy	• PHD	• Exo_endo_phos_2
	• Peptidase_A17	• PHO4	• Ets
	• MRI	• G-patch	• Flu_PA
	• TYA	• PhoH	• RdRP_1
	• Orbi_VP4	• EXS	• Mononeg_RNA_pol
	• EVI2A	• Exo_endo_phos	• Flavi_NS5
	• Peptidase_A2B	• Tctex-1	• Bromo_coat
	• ALIX_LYPXL_bnd	• MRVI1	• RdRP4
	• Reo_sigma1	• Pkinase_Tyr	• Peptidase_A3
	• TLV_coat	• DUF1725	• Flavi_glycop_C
	• DUF1011	• DUF2155	• BAF
	• HLH	• AA_permease_2	• RNA_replicase_B
	• Pkinase	• Cupin_8	• Birna_RdRp
	• Ras	• AA_permease_C	• Peptidase_C34
	• bZIP_1	• DUF4219	• BLVR
	• UQ_con		


*HMMs in the “other” category correspond to common functional motifs, non-retroviral proteins, or domains with unknown function.*

### Representative Endogenous Retroviral Sequences

As expected, BLAST identified more, but less diverse sequences, than HMMER in both the human genome and Repbase consensa. tBLASTn for HK2 PR matched every ERVK clade except HK10, but no other ERV. HMMER identified an RVP domain in every ERVK clade, and most other ERVs. The regions identified by each method overlap. tBLASTn for the *Phoenix* RVT_1 domain matched most ERVs. HMMER also identified an RVT_1 domain in most ERVs. The results of these methods mostly overlapped, with some notable differences. BLAST identified only part of the RVT_1 domain on ERVE, and the RVT_1 domain on ERV9 and ERVFc covers only part of the BLAST result. Each of the BLAST and HMMER results from ERVFb only partially overlapped. HMMER identified an RVT_1 domain on ERVT, but BLAST found no match. Both methods disproportionately detected HML2 loci; BLAST did so because the query sequences were HML2-derived, whereas HMMER did so because HML2 loci are more intact and therefore more easily discovered by LTRharvest.

**FIGURE 1:**
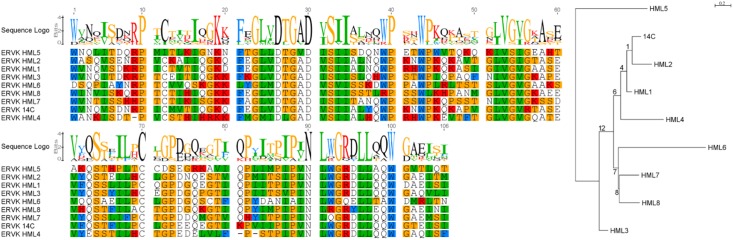
Sequences identified by tBLASTn which are similar to the HK2 protease. Representative ERV sequences from Repbase were searched using tBLASTn for the HK2 PR. These were aligned by MACSE and their phylogeny was inferred using RAxML under the JTT + G model of evolution selected using ProtTest 3. Bootstrap support values are represented as branch labels. This figure was produced using Geneious, FigTree, and GIMP.

The nucleotide sequences from each search were aligned using their translation and their phylogeny was inferred using RAxML from both nucleotide and amino acid (AA) alignments (see **Figure 1** for PR tBLASTn results). The resulting alignments and trees were used to classify the genomic sequences identified by the corresponding search. These reference trees have very low bootstrap support values; however, *pol* and *pro* trees have similar topology and the classifications based on evolutionary placement agree with the tree topology inferred directly from genomic *pro* sequences.

### Classification of ERV Sequences in the Human Genome

Having established a system to identify PR and RT elements, ERV nucleotide sequences were identified in GRCh38 using BLAST and HMMER as directed by GenomeTools. These were then curated to eliminate highly divergent sequences with rare insertions (less frequent than 1/20 sequences) and to minimize the size of their multiple alignment before phylogenetic inference. LTRdigest hits that did not co-occur with another retroviral domain were eliminated. The genomic distribution of the resulting ERV annotations is presented in **Table 2**, along with curation data.

**Table 2:** Distribution of PR and RT search results in the human genome GRCh38.

Chromosome	tBLASTn *ERVK-10* PR	LTRdigest RVP	tBLASTn *Phoenix* RVT_1		LTRdigest RVT_1	
				
			Raw	Curated	Raw	Curated
1	32	15	97	34	115	15
2	17	6	79	36	95	7
3	28	14	109	57	136	13
4	35	14	113	53	144	15
5	23	11	74	39	140	9
6	30	6	95	37	104	9
7	18	3	58	24	92	3
8	24	13	91	32	85	13
9	8	1	39	13	49	3
10	19	5	52	23	80	8
11	18	12	71	40	104	12
12	15	10	75	37	82	10
13	5	2	34	19	30	1
14	10	4	35	13	57	4
15	7	3	17	7	26	3
16	10	4	23	12	14	2
17	11	3	21	17	10	2
18	2	3	19	5	28	2
19	42	6	76	38	18	6
20	5	0	15	0	20	0
21	3	0	12	6	10	0
22	7	1	14	6	12	1
X	22	7	108	14	267	7
Y	37	7	85	10	48	4
Alternative	52	N/A	64	N/A	N/A	N/A
Total	480	150	1412	572	1766	149


*“Raw” refers to all sequences identified, and “Curated” refers only to sequences that were aligned for further analysis. Credible RT sequences are those which were not eliminated for causing gappy sites. Curated RVT_1 sequences are those which co-occurred with another retroviral HMM match. LTRharvest and LTRdigest were not used to search alternative assemblies, so there are no results to report. Since this curation workflow did not exclude any protease search results, the number is shown only once.*

When examining PR hits, tBLASTn for *ERVK-10* PR identified 480 sequences in GRCh38 (**Additional Files 1, 2**). Forty-two are identical to another PR sequence, of which 15 are from an alternative assembly. LTRdigest identified 150 RVP domains in GRCh38 (**Additional Files 3, 4**). Only 8 of these were shorter than half of the expected length of an RVP domain. Occasionally, sequences from different loci were indistinguishable; twelve were identical to another RVP locus. RVP positions 38–42 are absent in 83 PRs; 48 of these were confidently classified into one of ERVW, ERV9, ERVE, ERVT, or ERV3. The remaining 35 PR sequences were not confidently classified. In contrast, the PR sequences identified by LTRdigest are less numerous and more diverse than those identified by BLAST. There is a substantial overlap in the sequences recognized by each method, but LTRdigest identified HK2 PR with a disproportionately higher frequency than other ERVs, given their relative occurrence in the human genome (Gifford and Tristem, 2003; Bannert and Kurth, 2006).

When examining RT hits, tBLASTn for the RVT_1 domain of *Phoenix* identified 1412 sequences in GRCh38, of which 840 were removed because they induced gaps (**Additional Files 5, 6**). LTRdigest identified 1766 RVT_1 domains in GRCh38, of which 1617 were removed either because they did not co-occur with another core retroviral domain or they contained uncommon insertions which induced gaps in the alignment (**Additional Files 7, 8**). Standalone RVT_1 domains were eliminated. This is because non-ERV retroelements, such as LINEs (Boissinot et al., 2000), also encode a RT with an RVT_1 domain, but lack other retroviral proteins. These non-ERV retroelements would skew the analysis, since they are much more numerous; retroelements as a whole make up 42.2% of the human genome, but ERVs make up less than 8.3% [47]. Although LINEs are not flanked by LTRs, they are nonetheless annotated when LTRharvest mistakes other appropriately spaced repetitive sequences for LTRs.

In order to assign appropriate nomenclature to the identified sequences, we classified 489 loci (one or more PR or RT encoding regions separated by no more than 10,000 bp) (**Table 3**). We observe 53 loci contain only one BLAST or LTRdigest result, 324 loci contain 2, 57 loci contain 3, 53 loci contain 4, and one each contain 5 and 6 results. Four loci had all results placed on internal nodes by RAxML and were not classified at all. Eighteen loci contained sequences whose classification varied; in most cases these sequences were truncated or insertion of one retroelement inside another resulted in spurious association of sequences belonging to separate elements.

**Table 3:** Number of sequences classified into each category from each search method.

	PR		RVP		RT		RVT_1	
	
	NT	AA	NT	AA	NT	AA	NT	AA
ERVP					12	12	1	1
ERV9			34	31	114	114	22	22
ERVW			13	13	53	53	10	9
ERVH			24	22	62	61	11	9
ERVF					2	2		
ERVFXA					7	7		
ERVFB					1	1		
ERVRB			1		2	2		
ERVI					20	18		
ERVE			3	3	20	20	3	3
ERV3			2	2	2	2	1	1
ERVT			4	4			4	4
Gammaretrovirus total			81	75	283	280	51	48
HK1	39	39	1	1	16	16	2	2
HK2	62	60	32	20	53	53	36	35
HK4	7	7	2	2	6	6	2	2
HK9 (K14C)	15	12	2	2	5	5	2	2
HK10 (KC4)					5	1	1	
HK3	216	215	22	22	81	80	14	14
HK5	36	36			38	38	1	1
HK6	52	54			11	11		
HK7	3	3	2	2	10	10	7	7
HK8	34	35	8	8	35	34	17	16
Betaretrovirus total	464	461	69	57	260	254	82	79
ERVL					6	6	5	4
UNPLACED	16	19	0	18	10	19	10	17
INTERNAL					1	1		
Total	480		150		572		149


*PR, results from BLAST for HK2 protease; RT, results from BLAST for Phoenix RVT_1 domain of reverse transcriptase; RVP, results from LTRdigest for RVP domain; RVT_1, results from LTRdigest for RVT_1 domain; NT, results classified by their nucleic acid sequence; AA, results classified by their translation.*

Totals are shown for each ERV supergroup. The total number of unplaced sequences is also shown along with the number of sequences placed on internal branches, which in both cases were between the H/F and W/9 clades. Sequences were considered unplaced if the probability of their placement into the tree was less than 90%. One sequence was placed on an internal node outside these clades. AA alignments are translated from nucleotide (NT) alignments.

**FIGURE 2:**
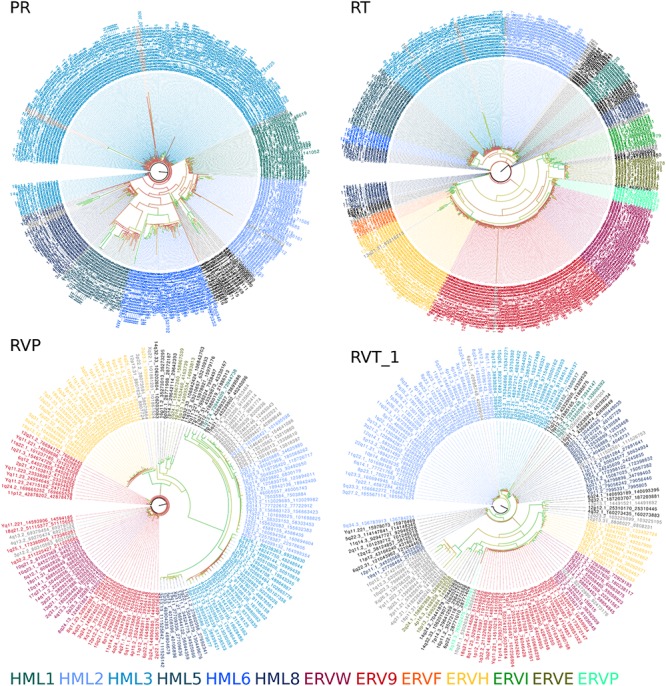
Amino acid sequence alignment derived phylogenies of human genomic endogenous retroviral sequences. A tree is shown for each search method (PR, protease BLAST; RT, reverse transcriptase BLAST; RVP, HMMER for RVP; RVT_1, HMMER for RVT_1). Leaves are colored by their classification through the evolutionary placement algorithm implemented by RAxML (see color legend at bottom of figure). Branch coloring represents bootstrap support, where red indicates poor support (closer to zero) and green indicates good support (closer to 100).

Phylogenetic trees constructed from genomic sequences (**Figure 2**) shared similar trends as those observed in the reference trees (**Figure 1** and **Additional Files 9–16**). The classifications assigned by placement of reference sequences into the phylogeny (**Additional Files 17–32**) agrees with the branching of the phylogeny inferred from genomic sequences directly. This can be clearly seen in **Figure 2**, in which the leaves of these phylogenies are colored according to their classification by evolutionary placement. Each phylogenetic tree (**Additional Files 33–40**) constructed from aligned nucleic acid sequences of either PR or RT from reference ERV sequences contained a bipartition segregating ERVK clades, although it should be noted that BLAST PR results include exclusively ERVK sequences. HK1, 2, 4, 9, and 10 commonly formed a clade within ERVK. In all four trees in **Figure 2**, ERVK leaves form a distinct clade, and likewise ERVW and ERV9 form a clade which is associated with ERVF. ERVS forms a clade with ERVL. ERVI forms a clade with ERVADP. ERVE forms a clade with ERV3. **Figure 2** is intended to allow the reader to quickly see the broad trends; if the reader is interested in the placement of specific elements, the phylogenetic trees output by RAxML are included in **Additional Files 33–40**.

### Alternate Genome Assemblies Reveal Additional PR Sequences

Although it fails to capture the real diversity of humanity, the reference human genome contains variable regions with multiple valid assemblies. New loci can be distinguished from loci which were duplicated by inclusion in both assemblies by examining the flanking genomic sequences, which should also be identical for technical duplicates. The 15 kilobases flanking the start site of each PR identified in GRCh38 by BLAST and GenomeTools was extracted and compared to alternative assemblies using blastn. Eight regions had a perfect match, of which five were originally identified by BLAST. Many BLAST hits surrounding identical PR sequences had partial matches indicative of closely similar insertions in divergent genomic loci. Moreover, 31 of the 52 regions identified on alternative assemblies had no high scoring matches. The fact that many sequences could not be paired up is consistent with previous observation of unique insertions in a recent analysis of the ERV content within alternative assemblies (Wildschutte et al., 2014).

### Diversity of Protease Sequences

**FIGURE 3:**
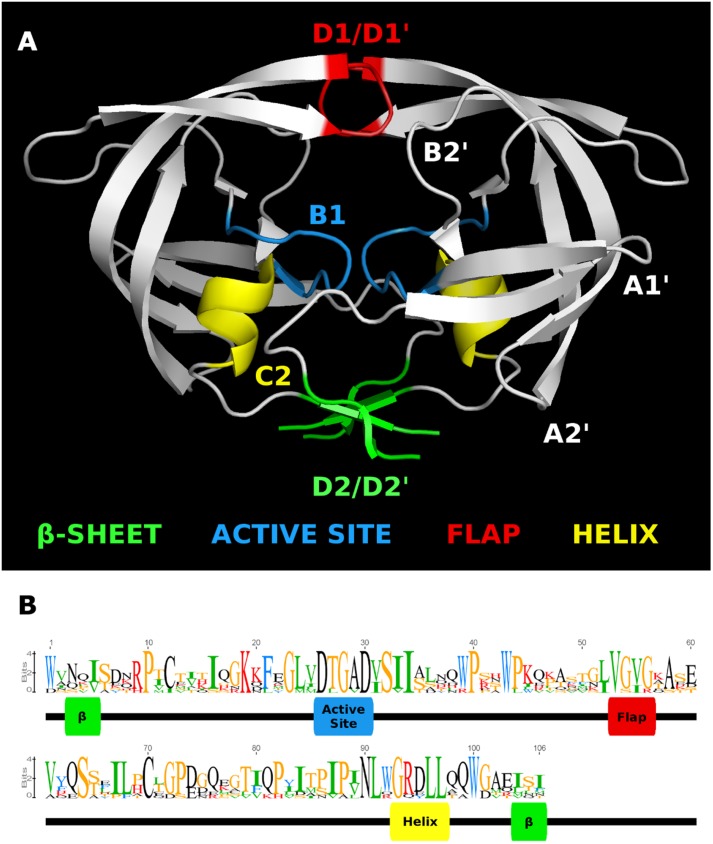
HMM logo of representative mature endogenous retrovirus K proteases. **(A)** The structure of the HIV-1 protease 3A2O (Hidaka et al., 2009) is shown with the major regions referenced in the text highlighted. **(B)** HMM logo of the aligned mature protease sequences of each ERVK entry in Repbase (HK1 through 10) with referenced regions highlighted in the same colors as the homologous regions in 3A2O. This figure was made with Pymol, Geneious, and GIMP.

**FIGURE 4:**
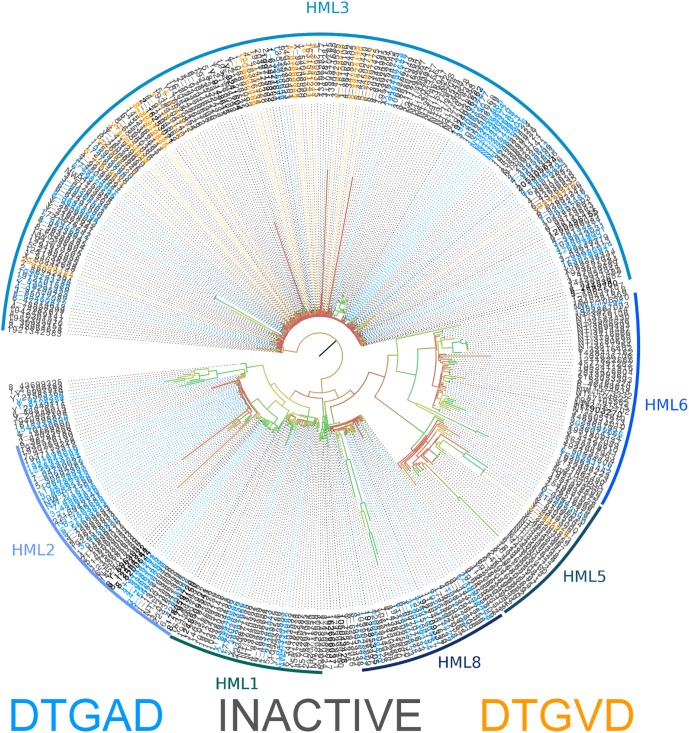
Phylogeny of human genomic nucleotide sequences derived using BLAST and the ERVK HK2 PR. Potentially active sequences with a DTGAD motif in their active site are colored blue, and those with DTGVD are colored orange. Sequences containing inactivating mutations are colored gray. The classification of each area of the tree is indicated by the name associated with the arc around the perimeter. Branch coloring represents bootstrap support, where red indicates poor support (closer to zero) and green indicates good support (closer to 100). Placement of the root at the branch connecting HK3 is only a matter of visual convenience – the real root branch is more likely that leading to HK8. This alignment is available in **Additional File 41**.

The consensus sequences for each ERVK clade from Repbase contain many conserved sites (**Figure 1**). It is clear that PR functional motifs are less diverse than the surrounding sequences (**Figure 3**), and that variants occur with different frequencies (**Table 4**). The α-helix (C2) has only one common variant (GRDLL). The active site loop (B1) has two; the DTGAD motif is most common and occurs in all clades, whereas the second most frequent (DTGVD) occurs only in the HK3, HK5, and HK6 clades. The tree in **Figure 4** is displayed to clearly show the abundance of HK3 sequences (top of figure) versus all other clades. Additionally, **Figure 4** highlights the distribution of common active site variants (DTGAD in blue, DTGVD in orange).

**Table 4:** Frequency of co-occurrence for observed ERVK active site and associated helix motifs.

Number	B1	C2	Number	B1	C2	Number	B1	C2
**Predicted active if translated**								
52	DTGAD	GRDLL	17	DTGVD	GRDLL			
**Potentially active**								
4	DTRAD	GRDLL	1	DTVVD	GRDLL	1	DTGDD	GRDLL
4	DTEAD	GRDLL	1	DTVAD	GRDLL	1	DTGAN	GRDLL
3	DTGSD	GRDLL	1	DTGVD	GRHLL	1	DTGAN	GKDLL
3	DTGED	GRDLL	1	DTGVD	GKDLL	1	DTGAD	GRELL
3	DTGAD	GQDLL	1	DTGTD	GRDLL	1	DTGAD	GRDIL
3	DTGAD	GKDLL	1	DTGMD	GRDLL	1	DTGAD	GQELL
3	DTDAD	GRDLL	1	DTGID	GRDLL	1	DTAAD	GRDLL
2	DTEVD	GRDLL	1	DTGGD	GRDLL			
**Predicted inactive if translated**								
4	DTGAD	RRDLL	1	DTRSD	GRDLL	1	DTGAD	GHLL
3	DTGAD	ERDLL	1	DTRMD	GKEIY	1	DTEAD	GQDLL
2	HTGAD	GRDLL	1	DTRAD	GWDPL	1	DRGMD	GRDLL
2	DTVVD	GGTLL	1	DTGVD	LSPHTFI	1	DMGAD	DQDLL
2	WAWAV	GWDLL	1	DTGVA	GRDLL	1	DMGAD	
1	VTGVD	GRDLL	1	DTGPD		1	DIGVD	GRDLL
1	ITWGR	GVDN	1	DTGVD	RRDLL	1	DIGAD	GGDLL
1		GWDLL	1	DTGAD	VWDLL	1	DIGAD	ERDLLL
1	ETGVD	GWDLL	1	DTGAD	GVDLL	1	DAGAD	GRDLL
1	DTVAD	GGDLL	1	DTGAD	GTRPI	1	ATGAD	GRDLL
1	DTRTD	GRDVL	1	DTGAD	GRYLL	1	ALGAD	RRDLL


Active site motifs were extracted from MACSE aligned protease sequences identified by BLAST in the human genome (GRCh38). Sequences containing stop codons and/or frameshift mutations were removed and their frequency was determined. This was done using standard UNIX utilities. Sequences were considered inactive if they contained mutations not observed in active proteases of other XRVs; otherwise, they were considered potentially active.

### ERVK HML2 Proteases Exhibit Great Diversity

**FIGURE 5:**
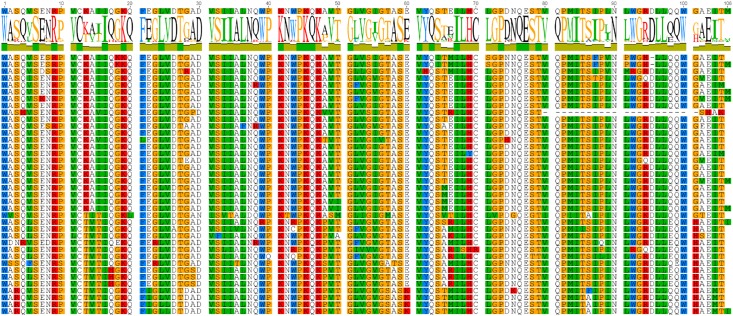
Aligned protein sequences of human genomic HML2 proteases. The subset of the full protein alignment of BLAST results for the ERVK HK2 PR in the human genome which were classified as HK2 by the evolutionary placement algorithm are shown here. Two clear patterns emerge, suggesting distinct protease sequence groups. Figure produced using Geneious.

Sequences classified as HK2 by RAxML were examined in more detail to determine if their variability could have functional consequences if the protease is translated, especially if it could lead to differential drug susceptibility because the variable residue intrudes into the substrate binding site. Several columns of the alignment in **Figure 5** represent variable sites which clearly subdivide the clade in two.

The most notable variation within HK2 sequences (**Figure 5**) is the variable AA (L, V or I) at position 89 which is predicted to help form the S3 and S1^′^ binding subsites by homology (Menendez-Arias et al., 1994). Columns 55, 65, and 66 are also notable. The AA (V or I) at position 55 follows the flap interface (columns 51–54) predicted by homology (Kuhelj et al., 2001). Residues 65 and 66 are near the surface of PR in the N-terminal strand of A2; from this position, their variability could impact protein-protein interactions within the viral polyproteins or with host partners, and could potentially influence flap mobility during substrate binding via interactions with α-helix C1. Thus, the cellular complement of HK2 PR variants represent a continuum of enzymatic affinities and protein-protein interactions.

### Variant Proteases Are Expressed in Human Health and Disease

**FIGURE 6:**
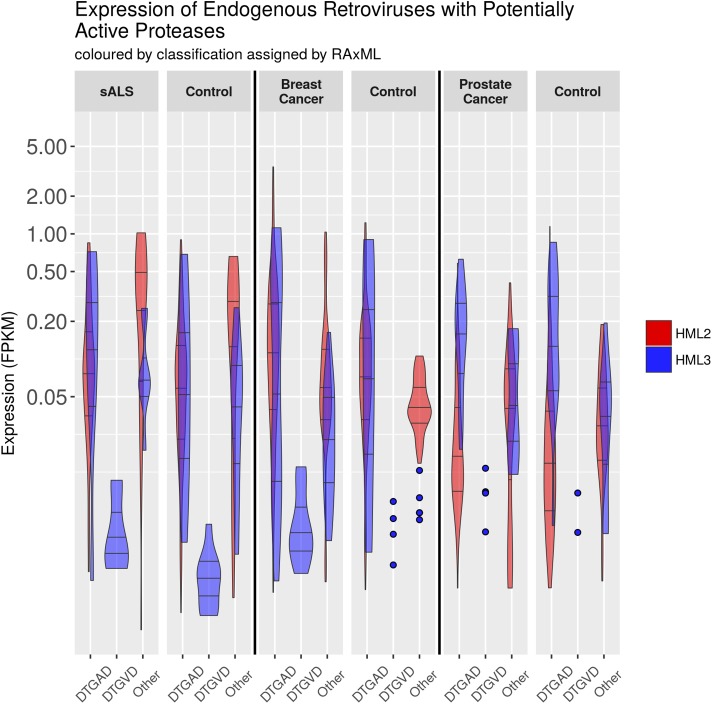
Expression of HML2 and HML3 by RNA-Seq. The expression of ERV loci in GRCh38 containing an uninterrupted protease was measured with bowtie2 using RNA-Seq data from different conditions (SRA accession numbers for each condition are: ALS – SRP064478, Breast Cancer – SRP058722, Prostate Cancer – ERP000550). Normalization to fragments per kilobase of exon per million mapped reads (FPKM) is relative to the entire locus. This graphic was made using R with the tidyverse library, and GIMP. The N’s of each column are, from left to right, HML2 and then HML3: 104, 8, 0, 7, 29, 7, 118, 9, 0, 10, 35, 8; 369, 27, 0, 17, 108, 25, 146, 11, 0, 4, 40, 5; 195, 14, 0, 4, 64, 13, 177, 14, 0, 2, 52, 13. Counts are also provided in CSV and Excel format in **Additional Files 44, 45**.

We looked in publicly available RNA-Seq datasets (**Table 5**) from diseases with some known association with ERV expression to establish how genomic diversity is transcribed in healthy and disease states (Seifarth et al., 2005; Douville et al., 2011; Ren et al., 2012; Agoni et al., 2013; Wildschutte et al., 2014; Abba et al., 2015; Li et al., 2015; Brohawn et al., 2016). RNA-Seq results were narrowed to examine the expression of the two most highly expressed and well-studied groups HML2 and HML3. Further, ERVK loci (detectable by both BLAST and LTRharvest as previously described) were limited to proteases without gross inactivating mutations. Reanalysis of transcriptomics data from three independent studies focused on ALS, breast cancer and prostate cancer are shown in **Figure 6**; expression of other HML groups are in **Additional File 42**. Overall, the expression of loci encoding DTGAD and DTGVD motifs is significantly different, due to low expression of DTGVD loci, and each of these is divergent from the pooled expression of other less common motifs.

**Table 5:** RNA-Seq Libraries from the Sequence Read Archive analyzed in this study.

Accession	Condition	Tissue	Library	Sequencing platform	Samples	Reference
SRP064478	sALS	Cervical spinal cord	Truseq Stranded Total RNA HT Kit (Illumina)	Nextseq 500 from ∼260 bp fragments	7 sALS, 8 non-ALS, without technical replication	Brohawn et al., 2016
ERP000550	Prostate cancer	Prostate	Oligo dT purification, fragmentation, and random hexamer PCR	HiSeq 2000 2 × 90 bp from ∼200 bp fragments	14 tumor/non-tumor from same patient, without technical replication	Ren et al., 2012
SRP058722	High grade ductal carcinoma *in situ*	Breast	ScriptSeq v2 RNA-Seq Kit (Epicenter)	HiSeq 2000 2 × 76 bp, fragment length unreported	25 ductal carcinoma *in situ*, 10 cultured primary normal breast tissue, without technical replication	Abba et al., 2015


Expression of protease encoding transcripts alone does not prove that these will be properly translated or proteolytically processed (Bauerova-Zabranska et al., 2005; Tien et al., 2018). Additional supporting evidence comes from examination of the upstream *gag* gene by searching reads aligning to the same locus using tBLASTn for the Gag sequence of ERV-K113. Several, but not all, loci expressed *gag* as well as *pro.* Individual experiments are required to confirm Gag-pro-pol polyprotein processing for each transcribed locus.

There is a trend toward increased ERVK transcription in cervical spinal tissues of ALS patients versus neuro-normal controls (**Figure 6**); indeed, this enhancement is evident when patients are stratified by sex, with female cases having an increased burden of ERVK (**Additional File 43**, *p* < 0.01). When tissues from cancer patients were examined, breast cancer biopsies revealed increased PR transcripts with DTGAD (*p* < 0.05) and alternate PR active site motifs (*p* < 0.05), as compared to cosmetic breast resection controls. Overall, HML-2 expression was enhanced in breast cancer (*p* < 0.01). This ERVK load difference in breast cancer appears to be driven by the increased expression of three loci in 8p23.1 flanked by LTR5A, and encoding a DTGSD active site motif. Interestingly, an HIV-1 PR variant which encodes a DTGSD motif is known to be active, although less so than the wild-type enzyme (Hong et al., 1998). Similarly, HML-2 transcripts are increased in prostate cancer specimens as compared to autologous non-cancerous adjacent tissue (*p* <0.01). These findings point toward a mixed profile of PR variants in disease states with elevated ERVK expression.

## Discussion

The human genome is home to a diversity of ERVs; this necessitates much effort in discriminating those of biological and/or clinical importance. Our identification of transcribed ERVK loci with a potential to encode functional PR enzymes will inform future studies examining their biological impact and drug susceptibility.

There remain technical challenges in assessing ERVs, as the results from LTRharvest depict that this paradigm is not ideal for analysis of more complicated loci containing recombination, serial insertions, or substantial deletions. Theoretically, each ERV should encode exactly 1 *pro* and 1 *pol* at the moment of insertion, but over time deletion and insertion events can remove or obscure these signatures. Despite this issue, the use of HMMs to identify ERVs within the human genome was effective. Indeed, RVP and RVT_1 annotations are more commonly identified within elements annotated by LTRharvest, since BLAST did not detect loci distantly related to the query HK2 sequences. The search results were improved by the merging of results from the same paralog and alignment-based extension. These sequences were grouped both by direct phylogenetic inference and by comparison to a reference tree. The phylogenetic patterns apparent in **Figures 1, 2** broadly agree with previously recognized relationships between ERVs (Vargiu et al., 2016). Although trees from *pro* and *pol* differ in some respects, they generally agree at deeper nodes which are important for classification, despite the lower phylogenetic signal of protease due to its comparatively shorter length.

There is value in examining both reference genomes and alternate assemblies. The reference genome was constructed from few people (Osoegawa et al., 1998; Lander et al., 2001), suggesting that the ERV content of individual human genomes could vary considerably and deserves closer scrutiny; especially since unfixed loci are likely to be younger (Belshaw et al., 2005), and could encode functional enzymes. Future use of data from platforms such as the 1000 Genomes Project (2018) will provide an improved appreciation of the diversity of ERV content in human DNA.

### ERVK Protease Sequence Diversity

The diversity of human genomic retroviral proteases reflects their evolutionary history and conserved structure-function relationships. Residues flanking the active site and those maintaining the dimer interface are less variable, a pattern clearly discernible in the Pfam family RVP (Finn et al., 2014) and evident within **Figure 1**. The β-sheet at the “bottom” of the enzyme is an exception to this rule, as its residues are not as conserved, perhaps because this region of tertiary structure is driven by backbone interactions (Louis et al., 2007). Overall, the most notable variation in genomic protease sequences is the absence of RVP positions 38–42 in 83 PRs, none of which were classified as ERVK.

The sequence of the retroviral protease active site (B1) and the active-site associate helix (C2) are conserved, as seen in **Figures 1, 3, 5** for ERVK. The most common active site motif of ERVK, DTGAD, is identical to that predominantly observed in exogenous retroviruses. The second most common motif, DTGVD (A29V), is observed in functional retroviral proteases; in fact, there are many examples of HIV proteases bearing this variation (e.g., Uniprot: P15833). Given the wide phylogenetic distribution of A29V and the similar chemical properties of alanine and valine residues, this is probably a functional variant of the ERVK protease. This motif could therefore represent pre-integration variation of the ancestral XRV which gave rise to ERVK, which is further supported by the concentration of DTGVD motif containing sequences in HK3, seen in **Figure 4**. Alternatively, the motif could represent a post-integration mutation predating the amplification of HK3 (Mayer and Meese, 2002). We also predict several α-helix (C2) variants could be functional in ERVK. In the HIV-1 PR (e.g., PDB: 1HVC), the C2 arginine residue (R94) fills the space between B1 and the loop connecting A1^′^ to D2^′^ while forming hydrogen bonds to residues in both. Another large polar residue might fill this role; indeed, lysine is known to do so in HIV-1 protease (Louis et al., 2007). Several genomic motifs fit these requirements, such as the HK2 R94Q and R94K variants, and the HK8 D95E variant. Systematic experimental verification of the activity of individual PR variants is thus warranted.

Nonetheless, we predict that most of the less common active site loop variants are inactive (**Figure 4**). This prediction is pulled from our understanding of the composition of this motif. Many variations are of a residue which catalyzes the hydrolytic reaction, or which is predicted to be essential to the dimer interface. The catalytic residues must be aspartic acid. The threonine residue which helps form the dimer interface is uncommonly replaced by serine (e.g., PDB: 2JYS, 2RSP) (Jaskolski et al., 1990; Hartl et al., 2008). Others could disrupt the active site by substituting a side-chain of very different size. The glycine residue must be small, as distortions to the structure of this region could disrupt the catalytic structure. This is also true for the alanine residue, although it is known to be replaced by serine in HIV-1 without a total loss of activity (Ido et al., 1991). The final aspartic acid residue participates in hydrogen bonds with the nearby arginine residues (Louis et al., 2007), and might be replaced by another small charged or polar residue; however, the character of this residue may not be critical, since it can vary with glycine in HIV-1 protease (Louis et al., 2007).

The young, polymorphic HK2 element ERVK113 is well studied and was reported to produce immature virions, perhaps due to a non-functional protease (Boller et al., 2008). This is in contrast to the known active HK2 protease of *ERVK-10* (Ono, 1986; Towler et al., 1998). This is further supported by reported differences in the sequences of these two mature proteases; the G56S (reported as G234S) substitution in ERVK113 may disrupt the flap interface, compromising PR activity and conferring an immature particle morphology (Boller et al., 2008).

### Implications of ERVK PR Diversity on Immunity

Retroviral proteases have numerous and varied protein targets which are difficult to predict, with correspondingly broad cellular effects stemming from their expression. Many proteases include targets with immune functions, but these vary based on host-virus pairings. It remains to be seen how ERVK PRs may modulate innate immunity signaling in humans, as seen with XRVs (Abudu et al., 2006; Solis et al., 2011; Wagner et al., 2015; Yoshikawa et al., 2017). Based on our knowledge of HIV (Konvalinka et al., 1995; Snasel et al., 2000; Lin et al., 2003), it is even conceivable that each ERVK PR variant may target distinct panels of cellular proteins, due to their different substrate specificities.

The potential impact of ERVK PR not limited to the innate immune system. Observations from the recovery of immune function in patients on HAART containing or not containing PIs suggests that HIV PR can directly impact the functioning of adaptive immune cells independently of other viral proteins (Ananworanich et al., 2003; Chiodi, 2006). Furthermore, differential ERVK PR substrate specificities could lead to changes in the peptide fragment population from which MHC class I epitopes are drawn, in addition to the impact that PI use alters this system (Kourjian et al., 2014; Kourjian et al., 2016). Our observation of distinct ERVK PR variants expressed in disease highlights the need for future studies to consider their overlapping and distinct impacts on cell signaling and proteome profiles related to immunity.

### Implications for Drug-Based Treatment of ERVK-Associated Disease

Many protease coding sequences could be translated into an active enzyme, and their sequence variability may impact their activity, and likely their susceptibility to antiretroviral drugs. Variation between DTGAD and DTGVD in the active site of sequences classified as HK3 is notable. This variation could have biological relevance when both HK2 and HK3 elements are expressed, such as in ALS (Douville et al., 2011) and schizophrenia (Frank et al., 2005). The HK2 PR is susceptible to some clinically relevant HIV-1 PIs, although not to the same degree as the HIV-1 PR (Towler et al., 1998; Kuhelj et al., 2001; Tyagi et al., 2017). Since ERVK expression is associated with many disease states, and the activity of retroviral enzymes can be pathogenic, it is possible that PIs may be useful in the future treatment of ERVK-associated conditions. In fact, at least one ongoing clinical trial (NCT02437110) plans to administer the PI Darunavir, along with other drugs, to patients with ALS. Another clinical trial (NCT01528865) targeting ERVK in lymphoma was withdrawn – but the intent to explore the clinical utility of ERVK PIs is becoming clear.

The rapid development of drug resistance is a major hurdle in the treatment of HIV-1 infections, but since ERVK is fixed in the host genome, it cannot rapidly evolve such drug resistance. However, ERVK elements are diverse and likely respond differently to PIs. This diversity is not represented in the ERVK literature, which is focused on the consensus HK2 PR (Towler et al., 1998; Kuhelj et al., 2001; Dewannieux et al., 2006; Lee and Bieniasz, 2007; Tyagi et al., 2017). One recent paper states that the active site loop of the HIV-1 and ERVK PR are identical (Tyagi et al., 2017), a finding which this report directly contradicts. We predict this diversity may necessitate the simultaneous use of multiple PIs in the treatment of ERVK-associated diseases. Consequently, if the drug regimen in the above-mentioned clinical trial is not effective in treating ALS, this cannot be taken as evidence that such therapy would not be effective using multiple PIs in a combination therapy.

We have established that a multitude of ERVK protease variants exist in the human genome, and that some of these variants can reasonably be predicted to have differing drug binding profiles. However, many ERVs are transcriptionally repressed (Bogerd et al., 2006; Schlesinger and Goff, 2013; Schlesinger et al., 2014); our analysis of publicly available RNA-Seq data shows not only that ERVs are expressed, but that the expression of loci with differing biochemical and evolutionary characteristics varies between and within different disease conditions, as well as in healthy controls. Due to the relatively recent insertion of ERVK sequences within the human genome, and the pathogenicity of XRV PRs, it is warranted that future studies consider their role in modulating immunity. Since distinct biochemical and ancestral features may impact the susceptibility of these enzymes to PIs, we propose that patient-specific drug regimens may be required in treatment of ERVK associated disease. Moreover, given the potential importance of protease sequence variability, the sequences of other ERVK proteins (particularly the drug targets reverse transcriptase and integrase) should also be explored.

## Conclusion

Within the human genome, ERVK proteases exhibit a high degree of diversity. Specifically, two predominant PR active sites emerge, the DTGAD and DTGVD variants, which are differentially expressed in disease states. This study will also be an asset for inferring how ERVK PRs impact the human proteome, specifically as it pertains to immune function. It is possible to interpret this paper as casting a negative light on the prospect of inhibitor-based treatment of ERVK-associated disease, but this is not our intention. We do, however, caution against treatments and techniques that treat the numerous and diverse elements called ERVK as though they were a single invariable element. This variability is not endless; biomedical and clinical techniques to account for this enzymatic variation exist. We recommend that the results of inhibition assays against the specific ERVK proteases expressed in each disease state should be considered if anti-PR drug regimens are to be implemented for inflammatory disease.

## Materials and Methods

Sources for databases and software employed in the course of this study are listed in **Table 6**.

**Table 6:** Databases and software used in this study.

Name	URL	Version or accession date
Bowtie2	https://www.github.com/BenLangmead/bowtie2	2.3.3
BLAST	ftp.ncbi.nlm.nih.gov/blast/executables/blast+/	2.2.28
Cytological bands	ftp.ncbi.nih.gov/genomes/MapView/Homo_sapiens/objects/	2015-11-20
FASTQC	https://www.bioinformatics.babraham.ac.uk/projects/fastqc/	0.11.5
Figtree	http://tree.bio.ed.ac.uk/software/figtree/	1.4.1
Geneious	https://www.geneious.com/	
GenomeTools	http://genometools.org/pub/	1.5.1
GIMP	http://www.gimp.org/downloads	2
GRCh38	ftp://ftp.ncbi.nlm.nih.gov/genomes/archive/old_genbank/Eukaryotes/vertebrates_mammals/Homo_sapiens/GRCh38/	2014-08-27
HMMER	http://hmmer.janelia.org	3.1b1
Htslib	https://github.com/samtools	1.5-22-g7a6854b
JAVA		1.8.0-144
jModelTest	(D Darriba) https://github.com/ddarriba/jmodeltest2	2
MACSE	(V Ranwez) http://www.mybiosoftware.com/macse-1-01b-multiple-alignment-of-coding-sequences.html	1.01b
Pfam-A	ftp.ebi.ac.uk/pub/databases/Pfam/current_release/Pfam-A.hmm.gz	2014-09-05
ProtTest	(D Darriba) https://www.github.com/ddarriba/prottest3	3
RAxML	https://github.com/stamatak/standard-RAxML	7.2.8
Repbase	(J Jurka) http://www.girinst.org/repbase/	19.07
RSYNC	https://rsync.samba.org/	v3.0.6 protocal v30
Samtools	https://github.com/samtools	1.5-9-g473d6a4
Sratoolkit	https://github.com/ncbi/sra-tools	2.8.2-1
Trimmomatic	https://github.com/timflutre/trimmomatic	0.36


Sequences homologous to retroviral protease (*pro*) and reverse transcriptase (*pol*) were automatically extracted from the last major build of the reference human genome and classified with published sequences from Repbase. Structure-function analysis was applied to ERV PR sequences using the limited published data on them, and by transferring knowledge of better studied proteases.

### Reference Sequences

The 38th official assembly of the human genome (GRCh38/hg38 published December 2013) was obtained from NCBI. Representatives for ERVADP, ERV9, ERV3, ERVE, ERVFRD, ERVF, ERVFXA, ERVFC, ERVFB, ERVH, ERVI, ERVL, ERVRB, ERVP, ERVS, ERVT, ERV16, ERVW, and HML groups 1 through 10 (HK1 through HK10) were derived from Repbase v19.07 (Jurka et al., 2005). HK9 and HK10 are also called K14C and KC4, respectively. The sequences of ERVK *Phoenix* (Dewannieux et al., 2006), and *ERVK-10* (Kuhelj et al., 2001) were taken from their respective publications. These sequences can be found in **Additional Files 46, 47**, respectively. Genes were located using BLAST v2.2.28 (Altschul et al., 1990) and HMMER v3.1b1 (Eddy, 1998). Cytological bands were assigned by a perl script using co-ordinates from NCBI.

### BLAST

Each GRCh38 chromosome and representative retroviral sequence was searched by tBLASTn for the amino acid sequences of the mature *ERVK-10* PR and the RVT_1 domain of *Phoenix pol* identified by HMMER. BLAST hits from same gene in different frames were merged, aligned by MACSE v1.01b (Ranwez et al., 2011), and then sequences 5^′^ and/or 3^′^ of each hit which would make the alignment flush were retrieved. Proteases identified in the reference genome assembly were matched to alternative assemblies using a perl script.

### HMMER and GenomeTools

HMMs for which to search were identified in Pfam-A using the search term “retrovirus” (Finn et al., 2014). ERV reference sequences were searched by HMMER with an *e*-value of 1.0. Putative LTR retroelements were identified in GRCh38 by LTRharvest, and these were subsequently analyzed by LTRdigest, which ran HMMER. LTRharvest and LTRdigest are part of Genometools v1.5.1 (Gremme et al., 2013). Hits from the same locus in different reading frames were merged and aligned with MACSE, then the regions which would make each sequence flush to the alignment were retrieved.

### Phylogenetic Inference and Classification

Results from each of the four searches were curated by eliminating HMM matches not associated with another core retroviral domain and by removing BLAST results which induced gaps in ≥95% of alignment rows with MACSE. Sequences were then re-aligned with MACSE and models of evolution were selected using jModelTest (Darriba et al., 2012) and ProtTest (Guindon et al., 2010; Darriba et al., 2011). Maximum likelihood phylogenies were inferred using RAxML v7.2.8 (Stamatakis, 2014) for nucleotide and protein alignments of each search (8 trees in total). GRCh38 results were classified using RAxML’s evolutionary placement algorithm with reference to the inferred phylogeny of representative ERV sequences.

Misclassified or recombinant elements were sought by comparing the assignment of search results separated by less than 10 kbp. This locus-based definition is computationally simple, but potentially error prone; a more accurate element-based analysis would require algorithmic reconstruction of insertion architecture, as was undertaken in the construction of Dfam (Wheeler et al., 2013).

### Measuring Expression in Publicly Available RNA-Seq Data

Publicly available RNA-Seq data from tissues associated with ERVK expression in human disease were obtained from the SRA using sratools. The resulting FASTQ files were aligned to the human genome (GRCh38) using bowtie2 following examination with FASTQC. The resulting SAM file was then indexed using samtools and finally the resulting indexed BAM file was queried for expression of ERV loci identified using the methods above. The raw expression values thus obtained were normalized by library size and locus length to produce FPKM values.

The resulting FPKM values were examined on density and QQ plots to confirm that they did not conform to a normal distribution. Non-parametric tests of statistical significance were used in this paper. For unpaired expression data (breast cancer, ALS) the Mann–Whitney *U* test was used, and for paired data (prostate cancer) the Wilcoxon Signed-Rank Test was used. In all cases tests were two-sided and *p*-values were corrected using the Bonferroni procedure, considering each set of contrasts involving the same variables to be a family.

## Availability of Data and Material

The datasets used to generate the findings of this paper are available from NCBI by their accession numbers. SRA accession numbers for each RNA-Seq data are: ALS – SRP064478, Breast Cancer – SRP058722, Prostate Cancer – ERP000550. The human genome version 38 was obtained from the NCBI FTP site [79]. Datasets generated during the current study which are not included as additional files are available from the corresponding author upon request. Important scripts used in the course of this study are included in **Additional File 48**.

## Author Contributions

MT and RD conceived the experiments. MT performed the bioinformatics analysis. MT and RD wrote the manuscript.

## Conflict of Interest Statement

The authors declare that the research was conducted in the absence of any commercial or financial relationships that could be construed as a potential conflict of interest.

## Supplementary Material

FILE 1 in FASTA (fa) format Aligned translated genomic protease BLAST results. The sequences found by BLAST for the HK2 protease in the human genome aligned and translated by MACSE.

FILE 2 in FASTA (fa) format Aligned genomic protease BLAST results. The sequences found by BLAST for the HK2 protease in the human genome aligned by MACSE.

FILE 3 in FASTA (fa) format Aligned translated genomic RVP HMMER results. The sequences found by HMMER directed by LTRdigest for the protein family HMM RVP in the human genome aligned and translated by MACSE.

FILE 4 in FASTA (fa) format Aligned genomic RVP HMMER results. The sequences found by HMMER directed by LTRdigest for the protein family HMM RVP in the human genome aligned by MACSE.

FILE 5 in FASTA (fa) format Aligned translated genomic reverse transcriptase BLAST results. The sequences found by BLAST for the HK2 reverse transcriptase in the human genome aligned and translated by MACSE.

FILE 6 in FASTA (fa) format Aligned genomic reverse transcriptase BLAST results. The sequences found by BLAST for the HK2 reverse transcriptase in the human genome aligned by MACSE.

FILE 7 in FASTA (fa) format Aligned translated genomic RVT_1 HMMER results. The sequences found by HMMER directed by LTRdigest for the protein family HMM RVT_1 in the human genome aligned and translated by MACSE.

FILE 8 in FASTA (fa) format Aligned genomic RVT_1 HMMER results. The sequences found by HMMER directed by LTRdigest for the protein family HMM RVT_1 in the human genome aligned by MACSE.

FILE 9 in Newick (nwk) format Phylogeny inferred from genomic BLAST results for the HK2 protease amino acid sequences. The phylogeny of amino acid sequences identified in the human genome GRCh38 by BLAST using the mature HK2 protease as a query as inferred by RAxML under the JTT + G model of evolution and aligned by MACSE.

FILE 10 in Newick (nwk) format Phylogeny inferred from genomic BLAST results for the HK2 protease nucleic acid sequences. The phylogeny of nucleic acid sequences identified in the human genome GRCh38 by BLAST using the mature HK2 protease as a query as inferred by RAxML under the GTR model of evolution and aligned by MACSE.

FILE 11 in Newick (nwk) format Phylogeny inferred from genomic BLAST results for the HK2 reverse transcriptase amino acid sequences. The phylogeny of amino acid sequences identified in the human genome GRCh38 by BLAST using the HK2 reverse transcriptase as a query as inferred by RAxML under the JTT + G model of evolution and aligned by MACSE.

FILE 12 in Newick (nwk) format Phylogeny inferred from genomic BLAST results for the HK2 reverse transcriptase nucleic acid sequences. The phylogeny of nucleic acid sequences identified in the human genome GRCh38 by BLAST using the HK2 reverse transcriptase as a query as inferred by RAxML under the GTR model of evolution and aligned by MACSE.

FILE 13 in Newick (nwk) format Phylogeny inferred from genomic HMMER results for RVP amino acid sequences. The phylogeny of amino acid sequences identified in the human genome GRCh38 by HMMER directed by LTRdigest using the protein family HMM RVP as a query as inferred by RAxML under the JTT + G model of evolution and aligned by MACSE.

FILE 14 in Newick (nwk) format Phylogeny inferred from genomic HMMER results for RVP nucleic acid sequences. The phylogeny of nucleic acid sequences identified in the human genome GRCh38 by HMMER directed by LTRdigest using the protein family HMM RVP as a query as inferred by RAxML under the GTR + G model of evolution and aligned by MACSE.

FILE 15 in Newick (nwk) format Phylogeny inferred from genomic HMMER results for RVT_1 amino acid sequences. The phylogeny of amino acid sequences identified in the human genome GRCh38 by HMMER directed by LTRdigest using the protein family HMM RVT_1 as a query as inferred by RAxML under the JTT + G model of evolution and aligned by MACSE.

FILE 16 in Newick (nwk) format Phylogeny inferred from genomic HMMER results for RVT_1 nucleic acid sequences. The phylogeny of nucleic acid sequences identified in the human genome GRCh38 by HMMER directed by LTRdigest using the protein family HMM RVT_1 as a query as inferred by RAxML under the GTR + G model of evolution and aligned by MACSE.

FILE 17 formatted as described in the RAxML user manual (txt) Classification likelihood weights for evolutionary placement of genomic protease BLAST amino acid sequences onto the corresponding reference tree. The placement of genomic sequences onto the tree of Repbase consensa was done using RAxML’s evolutionary placement algorithm under the JTT + G model of evolution, and this file contains the likelihood of each potential placement for each sequence.

FILE 18 formatted as described in the RAxML user manual (txt) Classification likelihood weights for evolutionary placement of genomic protease BLAST nucleic acid sequences onto the corresponding reference tree. The placement of genomic sequences onto the tree of Repbase consensa was done using RAxML’s evolutionary placement algorithm under the GTR + G model of evolution, and this file contains the likelihood of each potential placement for each sequence.

FILE 19 formatted as described in the RAxML user manual (txt) Classification likelihood weights for evolutionary placement of genomic reverse transcriptase BLAST amino acid sequences onto the corresponding reference tree. The placement of genomic sequences onto the tree of Repbase consensa was done using RAxML’s evolutionary placement algorithm under the RtREV + G model of evolution, and this file contains the likelihood of each potential placement for each sequence.

FILE 20 formatted as described in the RAxML user manual (txt) Classification likelihood weights for evolutionary placement of genomic reverse transcriptase BLAST nucleic acid sequences onto the corresponding reference tree. The placement of genomic sequences onto the tree of Repbase consensa was done using RAxML’s evolutionary placement algorithm under the GTR + G model.

FILE 21 formatted as described in the RAxML user manual (txt) Classification likelihood weights for evolutionary placement of genomic RVP HMMER amino acid sequences onto the corresponding reference tree. The placement of genomic sequences onto the tree of Repbase consensa was done using RAxML’s evolutionary placement algorithm under the WAG + G + F model of evolution, and this file contains the likelihood of each potential placement for each sequence.

FILE 22 formatted as described in the RAxML user manual (txt) Classification likelihood weights for evolutionary placement of genomic RVP HMMER nucleic acid sequences onto the corresponding reference tree. The placement of genomic sequences onto the tree of Repbase consensa was done using RAxML’s evolutionary placement algorithm under the GTR + G model of evolution, and this file contains the likelihood of each potential placement for each sequence.

FILE 23 formatted as described in the RAxML user manual (txt) Classification likelihood weights for evolutionary placement of genomic RVT_1 HMMER amino acid sequences onto the corresponding reference tree. The placement of genomic sequences onto the tree of Repbase consensa was done using RAxML’s evolutionary placement algorithm under the RtREV + G model of evolution, and this file contains the likelihood of each potential placement for each sequence.

FILE 24 formatted as described in the RAxML user manual (txt) Classification likelihood weights for evolutionary placement of genomic RVT_1 HMMER nucleic acid sequences onto the corresponding reference tree. The placement of genomic sequences onto the tree of Repbase consensa was done using RAxML’s evolutionary placement algorithm under the GTR + G model of evolution, and this file contains the likelihood of each potential placement for each sequence.

FILE 25 in Newick (nwk) format Original labeled reference tree for evolutionary placement of genomic protease BLAST amino acid sequences. The reference tree for placement of genomic protease BLAST results labeled for use with the corresponding classification likelihood file.

FILE 26 in Newick (nwk) format Original labeled reference tree for evolutionary placement of genomic protease BLAST nucleic acid sequences. The reference tree for placement of genomic protease BLAST results labeled for use with the corresponding classification likelihood file.

FILE 27 in Newick (nwk) format Original labeled reference tree for evolutionary placement of genomic reverse transcriptase BLAST amino acid sequences. The reference tree for placement of genomic reverse transcriptase BLAST results labeled for use with the corresponding classification likelihood file.

FILE 28 in Newick (nwk) format Original labeled reference tree for evolutionary placement of genomic reverse transcriptase BLAST nucleic acid sequences. The reference tree for placement of genomic reverse transcriptase BLAST results labeled for use with the corresponding classification likelihood file.

FILE 29 in Newick (nwk) format Original labeled reference tree for evolutionary placement of genomic RVP HMMER amino acid sequences. The reference tree for placement of genomic RVP HMMER directed by LTRdigest results labeled for use with the corresponding classification likelihood file.

FILE 30 in Newick (nwk) format Original labeled reference tree for evolutionary placement of genomic RVP HMMER nucleic acid sequences. The reference tree for placement of genomic RVP HMMER directed by LTRdigest results labeled for use with the corresponding classification likelihood file.

FILE 31 in Newick (nwk) format Original labeled reference tree for evolutionary placement of genomic RVT_1 HMMER amino acid sequences. The reference tree for placement of genomic RVT_1 HMMER directed by LTRdigest results labeled for use with the corresponding classification likelihood file.

FILE 32 in Newick (nwk) format Original labeled reference tree for evolutionary placement of genomic RVT_1 HMMER nucleic acid sequences. The reference tree for placement of genomic RVT_1 HMMER directed by LTRdigest results labeled for use with the corresponding classification likelihood file.

FILE 33 in Newick (nwk) format Phylogeny inferred from amino acid sequences from representative Repbase consensa searched using BLAST with the HK2 protease as a query. The phylogeny of amino acid sequences identified in representative Repbase consensa by BLAST using the mature HK2 protease as a query as inferred by RAxML under the JTT + G model of evolution and aligned by MACSE.

FILE 34 in Newick (nwk) format Phylogeny inferred from nucleic acid sequences from representative Repbase consensa searched using BLAST with the HK2 protease as a query. The phylogeny of nucleic acid sequences identified in representative Repbase consensa by BLAST using the mature HK2 protease as a query as inferred by RAxML under the GTR + G model of evolution and aligned by MACSE.

FILE 35 in Newick (nwk) format Phylogeny inferred from amino acid sequences from representative Repbase consensa searched using BLAST with the HK2 reverse transcriptase as a query. The phylogeny of amino acid sequences identified in representative Repbase consensa by BLAST using the HK2 reverse transcriptase as a query as inferred by RAxML under the RtREV + G model of evolution and aligned by MACSE.

FILE 36 in Newick (nwk) format Phylogeny inferred from nucleic acid sequences from representative Repbase consensa searched using BLAST with the HK2 reverse transcriptase as a query. The phylogeny of nucleic acid sequences identified in representative Repbase consensa by BLAST using the HK2 reverse transcriptase as a query as inferred by RAxML under the GTR + G model of evolution and aligned by MACSE.

FILE 37 in Newick (nwk) format Phylogeny inferred from amino acid sequences from representative Repbase consensa searched using HMMER for RVP. The phylogeny of amino acid sequences identified in representative Repbase consensa by HMMER using the protein family HMM RVP as a query as inferred by RAxML under the WAG + G + F model of evolution and aligned by MACSE.

FILE 38 in Newick (nwk) format Phylogeny inferred from nucleic acid sequences from representative Repbase consensa searched using HMMER for RVP. The phylogeny of nucleic acid sequences identified in representative Repbase consensa by HMMER using the protein family HMM RVP as a query as inferred by RAxML under the GTR + G model of evolution and aligned by MACSE.

FILE 39 in Newick (nwk) format Phylogeny inferred from amino acid sequences from representative Repbase consensa searched using HMMER for RVT_1. The phylogeny of amino acid sequences identified in representative Repbase consensa by HMMER using the protein family HMM RVT_1 as a query as inferred by RAxML under the RtREV + G + I model of evolution and aligned by MACSE.

FILE 40 in Newick (nwk) format Phylogeny inferred from nucleic acid sequences from representative Repbase consensa searched using HMMER for RVT_1. The phylogeny, inferred by RAxML under the GTR + G model of evolution, of nucleic acid sequences identified in representative Repbase consensa by HMMER using the protein family HMM RVT_1 as a query aligned by MACSE.

FILE 41 in FASTA (fa) format Alignment used to generate **Figure 4**.

FILE 42 in PNG (png) format Expression of non-HML2/HML3 ERVs in human tissues. The expression of ERVK loci in GRCh38 containing an uninterrupted protease was measured with bowtie2 using RNA-Seq data from different conditions (SRA accession numbers for each condition are ALS – SRP064478, Breast Cancer – SRP058722, Prostate Cancer – ERP000550). Normalization to fragments per kilobase of exon per million mapped reads (FPKM) is relative to the entire locus. This graphic was made using R with the tidyverse library, and GIMP.

FILE 43 in PNG (png) format Expression of ERVK in cervical spinal cord RNA-Seq of male and female donors with ALS. The expression of ERVK loci in GRCh38 containing an uninterrupted protease was measured with bowtie2 using RNA-Seq data from different conditions (SRA accession numbers for each condition are ALS – SRP064478, Breast Cancer – SRP058722, Prostate Cancer – ERP000550). Normalization to fragments per kilobase of exon per million mapped reads (FPKM) is relative to the entire locus. Samples were sexed using expression of XIST. This graphic was made using R with the tidyverse library, and GIMP.

FILE 44 in Comma Seperated Values (csv) format Counts of HML2/HML3 ERVs expressed in human tissues.

FILE 45 in Excel (xlsx) format Counts of HML2/HML3 ERVs expressed in human tissues.

FILE 46 in FASTA (fa) format Sequence of the HML2 protease. The sequence of the ERVK-10 mature protease.

FILE 47 in FASTA (fa) format Sequence of the HML2 reverse transcriptase. The sequence of the RVT_1 domain of the reconstituted HML2 progenitor virus Phoenix.

FILE 48 in ZIP (zip) format Important scripts used for this study. This ZIP archive contains the perl and bash scripts used to locate and align protease and reverse transcriptase using BLAST in the human genome and the scripts used to measure expression in RNA-Seq data.

## Abbreviations

AA: peptide/amino acid
ALS: amyotrophic lateral sclerosis
BLAST: basic local alignment search tool
CA: Capsid
DNA: desoxyribonucleic acid
dsRNA: double stranded RNA
DU: dUTPase
ERV: endogenous retrovirus
ERVK: endogenous retrovirus-K
FTP: file transfer protocol
GIMP: GNU image manipulation program
GRCh38: genome research consortium human genome release 38
GTR: general time-reversible
HIV: human immunodeficiency virus
HK: human endogenous retrovirus-K HML
HML: homology to mouse mammary tumor virus-like
HMM: hidden Markov model
IN: integrase
JTT: Jones, Taylor, and Thornton
LTR: long terminal repeat
MA: matrix
MACSE: multiple alignment of coding sequences
NCB: National Computational Biology Institute
NC: nucleocapsid
NT: nucleotide/nucleic acid
PI: protease inhibitor
PNG: portable network graphics
PR: protease
RAxML: randomized axelerated maximum likelihood
RH: RNAse H
RIG: retinoic acid inducible gene
RNA: ribonucleic acid
RNA-Seq: RNA sequencing
RT: reverse transcriptase
sALS: sporadic ALS
SRA: sequence read archive
ssRNA: single stranded RNA
SU: surface subunit of envelope
WAG: Whelan and Goldman
XIST: X inactive specific transcript
XRV: exogenous retrovirus

## References

[B1] 1000 Genomes Project (2018). Available at: http://www.internationalgenome.org/

[B2] Abba M. C. Gong T. Lu Y. Lee J. Zhong Y. Lacunza E. (2015). A molecular portrait of high-grade Ductal carcinoma *in situ*. *Cancer Res.* 75 3980–3990. 10.1158/0008-5472.CAN-15-0506 26249178 PMC4768486

[B3] Abudu A. Takaori-Kondo A. Izumi T. Shirakawa K. Kobayashi M. Sasada A. (2006). Murine retrovirus escapes from murine APOBEC3 via two distinct novel mechanisms. *Curr. Biol.* 16 1565–1570. 10.1016/j.cub.2006.06.055 16890533

[B4] Agoni L. Guha C. Lenz J. (2013). Detection of human endogenous retrovirus K (HERV-K) transcripts in human prostate cancer cell lines. *Front. Oncol.* 3:180. 10.3389/fonc.2013.00180 23847768 PMC3705622

[B5] Altschul S. F. Gish W. Miller W. Myers E. W. Lipman D. J. (1990). Basic local alignment search tool. *J. Mol. Biol.* 215 403–410. 10.1016/S0022-2836(05)80360-2 2231712

[B6] Ananworanich J. Nuesch R. Teeratakulpisarn S. Srasuebkul P. Chuenyam T. Siangphoe U. (2003). In vivo cell-mediated immunity in subjects with undetectable viral load on protease inhibitor-based versus non-protease inhibitor-based highly active antiretroviral therapy. *J. Acquir. Immune Defic. Syndr.* 32 570–572. 10.1097/00126334-200304150-00016 12679711

[B7] Bannert N. Kurth R. (2004). Retroelements and the human genome: new perspectives on an old relation. *Proc. Natl. Acad. Sci. U.S.A.* 101(Suppl. 2), 14572–14579. 10.1073/pnas.0404838101 15310846 PMC521986

[B8] Bannert N. Kurth R. (2006). The evolutionary dynamics of human endogenous retroviral families. *Annu. Rev. Genomics Hum. Genet.* 7 149–173. 10.1146/annurev.genom.7.080505.115700 16722807

[B9] Barbeau B. Mesnard J. M. (2015). Does chronic infection in retroviruses have a sense? *Trends Microbiol.* 23 367–375. 10.1016/j.tim.2015.01.009 25701112

[B10] Bauerova-Zabranska H. Stokrova J. Strisovsky K. Hunter E. Ruml T. Pichova I. (2005). The RNA binding G-patch domain in retroviral protease is important for infectivity and D-type morphogenesis of Mason-Pfizer monkey virus. *J. Biol. Chem.* 280 42106–42112. 10.1074/jbc.M508031200 16257973

[B11] Becker J. Perot P. Cheynet V. Oriol G. Mugnier N. Mommert M. (2017). A comprehensive hybridization model allows whole HERV transcriptome profiling using high density microarray. *BMC Genomics* 18:286. 10.1186/s12864-017-3669-7 28390408 PMC5385096

[B12] Belshaw R. Dawson A. L. Woolven-Allen J. Redding J. Burt A. Tristem M. (2005). Genomewide screening reveals high levels of insertional polymorphism in the human endogenous retrovirus family HERV-K(HML2): implications for present-day activity. *J. Virol.* 79 12507–12514. 10.1128/JVI.79.19.12507-12514.2005 16160178 PMC1211540

[B13] Bogerd H. P. Wiegand H. L. Hulme A. E. Garcia-Perez J. L. O’Shea K. S. Moran J. V. (2006). Cellular inhibitors of long interspersed element 1 and Alu retrotransposition. *Proc. Natl. Acad. Sci. U.S.A.* 103 8780–8785. 10.1073/pnas.0603313103 16728505 PMC1482655

[B14] Boissinot S. Chevret P. Furano A. V. (2000). L1 (LINE-1) retrotransposon evolution and amplification in recent human history. *Mol. Biol. Evol.* 17, 915–928. 10.1093/oxfordjournals.molbev.a026372 10833198

[B15] Boller K. Schonfeld K. Lischer S. Fischer N. Hoffmann A. Kurth R. (2008). Human endogenous retrovirus HERV-K113 is capable of producing intact viral particles. *J. Gen. Virol.* 89(Pt 2), 567–572. 10.1099/vir.0.83534-0 18198388

[B16] Bray S. Turnbull M. Hebert S. Douville R. N. (2016). Insight into the ERVK Integrase - Propensity for DNA Damage. *Front. Microbiol.* 7:1941. 10.3389/fmicb.2016.01941 27990140 PMC5131560

[B17] Brohawn D. G. O’Brien L. C. Bennett J. P. Jr. (2016). RNAseq analyses identify tumor necrosis factor-mediated inflammation as a major abnormality in ALS spinal cord. *PLoS One* 11:e0160520. 10.1371/journal.pone.0160520 27487029 PMC4972368

[B18] Chiodi F. (2006). A link between immune hyperactivation of T cells during HIV-1 infection and the virus protease? *AIDS* 20 769–771. 10.1097/01.aids.0000216378.84313.b6 16514308

[B19] Christensen T. (2016). Human endogenous retroviruses in neurologic disease. *APMIS* 124 116–126. 10.1111/apm.12486 26818266

[B20] Contreras-Galindo R. Kaplan M. H. Contreras-Galindo A. C. Gonzalez-Hernandez M. J. Ferlenghi I. Giusti F. (2012). Characterization of human endogenous retroviral elements in the blood of HIV-1-infected individuals. *J. Virol.* 86 262–276. 10.1128/JVI.00602-11 22031938 PMC3255917

[B21] Cowley M. Oakey R. J. (2013). Transposable elements re-wire and fine-tune the transcriptome. *PLoS Genet.* 9:e1003234. 10.1371/journal.pgen.1003234 23358118 PMC3554611

[B22] Darriba D. Taboada G. L. Doallo R. Posada D. (2011). ProtTest 3: fast selection of best-fit models of protein evolution. *Bioinformatics* 27 1164–1165. 10.1093/bioinformatics/btr088 21335321 PMC5215816

[B23] Darriba D. Taboada G. L. Doallo R. Posada D. (2012). jModelTest 2: more models, new heuristics and parallel computing. *Nat. Methods* 9:772. 10.1038/nmeth.2109 22847109 PMC4594756

[B24] Denner J. Young P. R. (2013). Koala retroviruses: characterization and impact on the life of koalas. *Retrovirology* 10:108. 10.1186/1742-4690-10-108 24148555 PMC4016316

[B25] Dewannieux M. Harper F. Richaud A. Letzelter C. Ribet D. Pierron G. (2006). Identification of an infectious progenitor for the multiple-copy HERV-K human endogenous retroelements. *Genome Res.* 16 1548–1556. 10.1101/gr.5565706 17077319 PMC1665638

[B26] Douville R. Liu J. Rothstein J. Nath A. (2011). Identification of active loci of a human endogenous retrovirus in neurons of patients with amyotrophic lateral sclerosis. *Ann. Neurol.* 69 141–151. 10.1002/ana.22149 21280084 PMC3052883

[B27] Dunn B. M. Goodenow M. M. Gustchina A. Wlodawer A. (2002). Retroviral proteases. *Genome Biol.* 3:REVIEWS3006 10.1186/gb-2002-3-4-reviews3006 PMC139352 11983066

[B28] Eddy S. R. (1998). Profile hidden Markov models. *Bioinformatics* 14 755–763. 10.1093/bioinformatics/14.9.755 9918945

[B29] Finn R. D. Bateman A. Clements J. Coggill P. Eberhardt R. Y. Eddy S. R. (2014). Pfam: the protein families database. *Nucleic Acids Res.* 42 D222–D230. 10.1093/nar/gkt1223 24288371 PMC3965110

[B30] Frank O. Giehl M. Zheng C. Hehlmann R. Leib-Mosch C. Seifarth W. (2005). Human endogenous retrovirus expression profiles in samples from brains of patients with schizophrenia and bipolar disorders. *J. Virol.* 79 10890–10901. 10.1128/JVI.79.17.10890-10901.2005 16103141 PMC1193590

[B31] Franklin G. C. Chretien S. Hanson I. M. Rochefort H. May F. E. Westley B. R. (1988). Expression of human sequences related to those of mouse mammary tumor virus. *J. Virol.* 62 1203–1210. 2831381 10.1128/jvi.62.4.1203-1210.1988 PMC253128

[B32] Gifford R. Tristem M. (2003). The evolution, distribution and diversity of endogenous retroviruses. *Virus Genes* 26 291–315. 10.1023/A:1024455415443 12876457

[B33] Gremme G. Steinbiss S. Kurtz S. (2013). GenomeTools: a comprehensive software library for efficient processing of structured genome annotations. *IEEE/ACM Trans. Comput. Biol. Bioinform.* 10 645–656. 10.1109/TCBB.2013.68 24091398

[B34] Grow E. J. Flynn R. A. Chavez S. L. Bayless N. L. Wossidlo M. Wesche D. J. (2015). Intrinsic retroviral reactivation in human preimplantation embryos and pluripotent cells. *Nature* 522 221–225. 10.1038/nature14308 25896322 PMC4503379

[B35] Guindon S. Dufayard J. F. Lefort V. Anisimova M. Hordijk W. Gascuel O. (2010). New algorithms and methods to estimate maximum-likelihood phylogenies: assessing the performance of PhyML 3.0. *Syst. Biol.* 59 307–321. 10.1093/sysbio/syq010 20525638

[B36] Hartl M. J. Wohrl B. M. Rosch P. Schweimer K. (2008). The solution structure of the simian foamy virus protease reveals a monomeric protein. *J. Mol. Biol.* 381 141–149. 10.1016/j.jmb.2008.05.064 18597783

[B37] Hidaka K. Kimura T. Abdel-Rahman H. M. Nguyen J. T. McDaniel K. F. Kohlbrenner W. E. (2009). Small-sized human immunodeficiency virus type-1 protease inhibitors containing allophenylnorstatine to explore the S2’ pocket. *J. Med. Chem.* 52 7604–7617. 10.1021/jm9005115 19954246

[B38] Holmes E. C. (2011). The evolution of endogenous viral elements. *Cell Host Microbe* 10 368–377. 10.1016/j.chom.2011.09.002 22018237 PMC7172163

[B39] Hong L. Hartsuck J. A. Foundling S. Ermolieff J. Tang J. (1998). Active-site mobility in human immunodeficiency virus, type 1, protease as demonstrated by crystal structure of A28S mutant. *Protein Sci.* 7 300–305. 10.1002/pro.5560070209 9521105 PMC2143907

[B40] Ido E. Han H. P. Kezdy F. J. Tang J. (1991). Kinetic studies of human immunodeficiency virus type 1 protease and its active-site hydrogen bond mutant A28S. *J. Biol. Chem.* 266 24359–24366. 1761538

[B41] Impens F. Timmerman E. Staes A. Moens K. Arien K. K. Verhasselt B. (2012). A catalogue of putative HIV-1 protease host cell substrates. *Biol. Chem.* 393 915–931. 10.1515/hsz-2012-0168 22944692

[B42] Jaskolski M. Miller M. Rao J. K. Leis J. Wlodawer A. (1990). Structure of the aspartic protease from Rous sarcoma retrovirus refined at 2-A resolution. *Biochemistry* 29 5889–5898. 10.1021/bi00477a002 2166563

[B43] Jha A. R. Nixon D. F. Rosenberg M. G. Martin J. N. Deeks S. G. Hudson R. R. (2011). Human endogenous retrovirus K106 (HERV-K106) was infectious after the emergence of anatomically modern humans. *PLoS One* 6:e20234. 10.1371/journal.pone.0020234 21633511 PMC3102101

[B44] Jurka J. Kapitonov V. V. Pavlicek A. Klonowski P. Kohany O. Walichiewicz J. (2005). Repbase Update, a database of eukaryotic repetitive elements. *Cytogenet. Genome Res.* 110 462–467. 10.1159/000084979 16093699

[B45] Kaneko-Ishino T. Ishino F. (2012). The role of genes domesticated from LTR retrotransposons and retroviruses in mammals. *Front. Microbiol.* 3:262. 10.3389/fmicb.2012.00262 22866050 PMC3406341

[B46] Kohl N. E. Emini E. A. Schleif W. A. Davis L. J. Heimbach J. C. Dixon R. A. (1988). Active human immunodeficiency virus protease is required for viral infectivity. *Proc. Natl. Acad. Sci. U.S.A.* 85 4686–4690. 10.1073/pnas.85.13.4686 3290901 PMC280500

[B47] Konvalinka J. Litterst M. A. Welker R. Kottler H. Rippmann F. Heuser A. M. (1995). An active-site mutation in the human immunodeficiency virus type 1 proteinase (PR) causes reduced PR activity and loss of PR-mediated cytotoxicity without apparent effect on virus maturation and infectivity. *J. Virol.* 69 7180–7186. 7474139 10.1128/jvi.69.11.7180-7186.1995 PMC189639

[B48] Kourjian G. Rucevic M. Berberich M. J. Dinter J. Wambua D. Boucau J. (2016). HIV protease inhibitor-induced cathepsin modulation alters antigen processing and cross-presentation. *J. Immunol.* 196 3595–3607. 10.4049/jimmunol.1600055 27009491 PMC4868670

[B49] Kourjian G. Xu Y. Mondesire-Crump I. Shimada M. Gourdain P. Le Gall S. (2014). Sequence-specific alterations of epitope production by HIV protease inhibitors. *J. Immunol.* 192 3496–3506. 10.4049/jimmunol.1302805 24616479 PMC3983674

[B50] Kozak C. A. (2014). Origins of the endogenous and infectious laboratory mouse gammaretroviruses. *Viruses* 7 1–26. 10.3390/v7010001 25549291 PMC4306825

[B51] Kraus B. Boller K. Reuter A. Schnierle B. S. (2011). Characterization of the human endogenous retrovirus K Gag protein: identification of protease cleavage sites. *Retrovirology* 8:21. 10.1186/1742-4690-8-21 21429186 PMC3073897

[B52] Kuhelj R. Rizzo C. J. Chang C. H. Jadhav P. K. Towler E. M. Korant B. D. (2001). Inhibition of human endogenous retrovirus-K10 protease in cell-free and cell-based assays. *J. Biol. Chem.* 276 16674–16682. 10.1074/jbc.M008763200M008763200 11278433

[B53] Lander E. S. Linton L. M. Birren B. Nusbaum C. Zody M. C. Baldwin J. (2001). Initial sequencing and analysis of the human genome. *Nature* 409 860–921. 10.1038/35057062 11237011

[B54] Lee Y. N. Bieniasz P. D. (2007). Reconstitution of an infectious human endogenous retrovirus. *PLoS Pathog.* 3:e10. 10.1371/journal.ppat.0030010 17257061 PMC1781480

[B55] Li W. Lee M. H. Henderson L. Tyagi R. Bachani M. Steiner J. (2015). Human endogenous retrovirus-K contributes to motor neuron disease. *Sci. Transl. Med.* 7:307ra153. 10.1126/scitranslmed.aac8201 26424568 PMC6344353

[B56] Lin Y. C. Beck Z. Morris G. M. Olson A. J. Elder J. H. (2003). Structural basis for distinctions between substrate and inhibitor specificities for feline immunodeficiency virus and human immunodeficiency virus proteases. *J. Virol.* 77 6589–6600. 10.1128/JVI.77.12.6589-6600.2003 12767979 PMC156162

[B57] Louis J. M. Ishima R. Torchia D. A. Weber I. T. (2007). HIV-1 protease: structure, dynamics, and inhibition. *Adv. Pharmacol.* 55 261–298. 10.1016/S1054-3589(07)55008-8 17586318

[B58] Macfarlan T. S. Gifford W. D. Driscoll S. Lettieri K. Rowe H. M. Bonanomi D. (2012). Embryonic stem cell potency fluctuates with endogenous retrovirus activity. *Nature* 487 57–63. 10.1038/nature11244 22722858 PMC3395470

[B59] Manghera M. Ferguson J. Douville R. (2014). Endogenous retrovirus-K and nervous system diseases. *Curr. Neurol. Neurosci. Rep.* 14:488. 10.1007/s11910-014-0488-y 25138026

[B60] Manghera M. Ferguson J. Douville R. (2015). ERVK polyprotein processing and reverse transcriptase expression in human cell line models of neurological disease. *Viruses* 7 320–332. 10.3390/v7010320 25609305 PMC4306841

[B61] Mayer J. Blomberg J. Seal R. L. (2011). A revised nomenclature for transcribed human endogenous retroviral loci. *Mob DNA* 2:7. 10.1186/1759-8753-2-7 21542922 PMC3113919

[B62] Mayer J. Meese E. U. (2002). The human endogenous retrovirus family HERV-K(HML-3). *Genomics* 80 331–343. 10.1006/geno.2002.6839 12213204

[B63] Menendez-Arias L. Weber I. T. Soss J. Harrison R. W. Gotte D. Oroszlan S. (1994). Kinetic and modeling studies of subsites S4-S3’ of Moloney murine leukemia virus protease. *J. Biol. Chem.* 269 16795–16801. 8207003

[B64] Mi S. Lee X. Li X. Veldman G. M. Finnerty H. Racie L. (2000). Syncytin is a captive retroviral envelope protein involved in human placental morphogenesis. *Nature* 403 785–789. 10.1038/35001608 10693809

[B65] Michaud H. A. de Mulder M. SenGupta D. Deeks S. G. Martin J. N. Pilcher C. D. (2014). Trans-activation, post-transcriptional maturation, and induction of antibodies to HERV-K (HML-2) envelope transmembrane protein in HIV-1 infection. *Retrovirology* 11:10. 10.1186/1742-4690-11-10 24472118 PMC3907665

[B66] Morandi E. Tarlinton R. E. Gran B. (2015). Multiple sclerosis between genetics and infections: human endogenous retroviruses in monocytes and macrophages. *Front. Immunol.* 6:647. 10.3389/fimmu.2015.00647 26734011 PMC4689809

[B67] Mueller-Lantzsch N. Sauter M. Weiskircher A. Kramer K. Best B. Buck M. (1993). Human endogenous retroviral element K10 (HERV-K10) encodes a full-length gag homologous 73-kDa protein and a functional protease. *AIDS Res. Hum. Retroviruses* 9 343–350. 10.1089/aid.1993.9.343 8512750

[B68] Naveira H. Bello X. Abal-Fabeiro J. L. Maside X. (2014). Evidence for the persistence of an active endogenous retrovirus (ERVE) in humans. *Genetica* 142 451–460. 10.1007/s10709-014-9789-y 25192754

[B69] Ono M. (1986). Molecular cloning and long terminal repeat sequences of human endogenous retrovirus genes related to types A and B retrovirus genes. *J. Virol.* 58 937–944. 3009897 10.1128/jvi.58.3.937-944.1986 PMC253002

[B70] Osoegawa K. Woon P. Y. Zhao B. Frengen E. Tateno M. Catanese J. J. (1998). An improved approach for construction of bacterial artificial chromosome libraries. *Genomics* 52 1–8. 10.1006/geno.1998.5423 9740665

[B71] Prudencio M. Gonzales P. K. Cook C. N. Gendron T. F. Daughrity L. M. Song Y. (2017). Repetitive element transcripts are elevated in the brain of C9orf72 ALS/FTLD patients. *Hum. Mol. Genet.* 26 3421–3431. 10.1093/hmg/ddx233 28637276 PMC5886204

[B72] Ranwez V. Harispe S. Delsuc F. Douzery E. J. (2011). MACSE: multiple alignment of coding SEquences accounting for frameshifts and stop codons. *PLoS One* 6:e22594. 10.1371/journal.pone.0022594 21949676 PMC3174933

[B73] Ren S. Peng Z. Mao J. H. Yu Y. Yin C. Gao X. (2012). RNA-seq analysis of prostate cancer in the Chinese population identifies recurrent gene fusions, cancer-associated long noncoding RNAs and aberrant alternative splicings. *Cell Res.* 22 806–821. 10.1038/cr.2012.30 22349460 PMC3343650

[B74] Reynier F. Verjat T. Turrel F. Imbert P. E. Marotte H. Mougin B. (2009). Increase in human endogenous retrovirus HERV-K (HML-2) viral load in active rheumatoid arthritis. *Scand. J. Immunol.* 70 295–299. 10.1111/j.1365-3083.2009.02271.x 19703019

[B75] Ruda V. M. Akopov S. B. Trubetskoy D. O. Manuylov N. L. Vetchinova A. S. Zavalova L. L. (2004). Tissue specificity of enhancer and promoter activities of a HERV-K(HML-2) LTR. *Virus Res.* 104 11–16. 10.1016/j.virusres.2004.02.036 15177887

[B76] Rumlova M. Krizova I. Keprova A. Hadravova R. Dolezal M. Strohalmova K. (2014). HIV-1 protease-induced apoptosis. *Retrovirology* 11:37. 10.1186/1742-4690-11-37 24886575 PMC4229777

[B77] Sauter M. Schommer S. Kremmer E. Remberger K. Dolken G. Lemm I. (1995). Human endogenous retrovirus K10: expression of Gag protein and detection of antibodies in patients with seminomas. *J. Virol.* 69 414–421. 7983737 10.1128/jvi.69.1.414-421.1995 PMC188589

[B78] Schlesinger S. Goff S. P. (2013). Silencing of proviruses in embryonic cells: efficiency, stability and chromatin modifications. *EMBO Rep.* 14 73–79. 10.1038/embor.2012.182 23154467 PMC3537137

[B79] Schlesinger S. Meshorer E. Goff S. P. (2014). Asynchronous transcriptional silencing of individual retroviral genomes in embryonic cells. *Retrovirology* 11:31. 10.1186/1742-4690-11-31 24742368 PMC4021621

[B80] Seifarth W. Frank O. Zeilfelder U. Spiess B. Greenwood A. D. Hehlmann R. (2005). Comprehensive analysis of human endogenous retrovirus transcriptional activity in human tissues with a retrovirus-specific microarray. *J. Virol.* 79 341–352. 10.1128/JVI.79.1.341-352.2005 15596828 PMC538696

[B81] Shoeman R. L. Kesselmier C. Mothes E. Honer B. Traub P. (1991). Non-viral cellular substrates for human immunodeficiency virus type 1 protease. *FEBS Lett.* 278 199–203. 10.1016/0014-5793(91)80116-K 1991513

[B82] Snasel J. Shoeman R. Horejsi M. Hruskova-Heidingsfeldova O. Sedlacek J. Ruml T. (2000). Cleavage of vimentin by different retroviral proteases. *Arch. Biochem. Biophys.* 377 241–245. 10.1006/abbi.2000.1776 10845700

[B83] Solis M. Nakhaei P. Jalalirad M. Lacoste J. Douville R. Arguello M. (2011). RIG-I-mediated antiviral signaling is inhibited in HIV-1 infection by a protease-mediated sequestration of RIG-I. *J. Virol.* 85 1224–1236. 10.1128/JVI.01635-10 21084468 PMC3020501

[B84] Stamatakis A. (2014). RAxML version 8: a tool for phylogenetic analysis and post-analysis of large phylogenies. *Bioinformatics* 30 1312–1313. 10.1093/bioinformatics/btu033 24451623 PMC3998144

[B85] Stewart C. Kural D. Stromberg M. P. Walker J. A. Konkel M. K. Stutz A. M. (2011). A comprehensive map of mobile element insertion polymorphisms in humans. *PLoS Genet.* 7:e1002236. 10.1371/journal.pgen.1002236 21876680 PMC3158055

[B86] Tien C. Huang L. Watanabe S. M. Speidel J. T. Carter C. A. Chen C. (2018). Context-dependent autoprocessing of human immunodeficiency virus type 1 protease precursors. *PLoS One* 13:e0191372. 10.1371/journal.pone.0191372 29338056 PMC5770051

[B87] Towler E. M. Gulnik S. V. Bhat T. N. Xie D. Gustschina E. Sumpter T. R. (1998). Functional characterization of the protease of human endogenous retrovirus, K10: can it complement HIV-1 protease? *Biochemistry* 37, 17137–17144. 10.1021/bi9818927 9860826

[B88] Turner G. Barbulescu M. Su M. Jensen-Seaman M. I. Kidd K. K. Lenz J. (2001). Insertional polymorphisms of full-length endogenous retroviruses in humans. *Curr. Biol.* 11 1531–1535. 10.1016/S0960-9822(01)00455-9 11591322

[B89] Tyagi R. Li W. Parades D. Bianchet M. A. Nath A. (2017). Inhibition of human endogenous retrovirus-K by antiretroviral drugs. *Retrovirology* 14:21. 10.1186/s12977-017-0347-4 28330477 PMC5361811

[B90] Vargiu L. Rodriguez-Tome P. Sperber G. O. Cadeddu M. Grandi N. Blikstad V. (2016). Classification and characterization of human endogenous retroviruses; mosaic forms are common. *Retrovirology* 13:7. 10.1186/s12977-015-0232-y 26800882 PMC4724089

[B91] Wagner R. N. Reed J. C. Chanda S. K. (2015). HIV-1 protease cleaves the serine-threonine kinases RIPK1 and RIPK2. *Retrovirology* 12:74. 10.1186/s12977-015-0200-6 26297639 PMC4546280

[B92] Wheeler T. J. Clements J. Eddy S. R. Hubley R. Jones T. A. Jurka J. (2013). Dfam: a database of repetitive DNA based on profile hidden Markov models. *Nucleic Acids Res.* 41 D70–D82. 10.1093/nar/gks1265 23203985 PMC3531169

[B93] Wildschutte J. H. Ram D. Subramanian R. Stevens V. L. Coffin J. M. (2014). The distribution of insertionally polymorphic endogenous retroviruses in breast cancer patients and cancer-free controls. *Retrovirology* 11:62. 10.1186/s12977-014-0062-310.1186/PREACCEPT-1720768941312026 25112280 PMC4149278

[B94] Wildschutte J. H. Williams Z. H. Montesion M. Subramanian R. P. Kidd J. M. Coffin J. M. (2016). Discovery of unfixed endogenous retrovirus insertions in diverse human populations. *Proc. Natl. Acad. Sci. U.S.A.* 113 E2326–E2334. 10.1073/pnas.1602336113 27001843 PMC4843416

[B95] Yoshikawa R. Takeuchi J. S. Yamada E. Nakano Y. Misawa N. Kimura Y. (2017). Feline immunodeficiency virus evolutionarily acquires two proteins, Vif and protease, capable of antagonizing feline APOBEC3. *J. Virol.* 91:e00250-17. 10.1128/JVI.00250-17 28331087 PMC5432859

