# Supplementary figures and images for: Related Endogenous Retrovirus-K Elements Harbor Distinct Protease Active Site Motifs
Source: Front Microbiol. 2018 Jul 18;9:1577. doi: 10.3389/fmicb.2018.01577 (PMC6058741; doi:10.3389/fmicb.2018.01577)

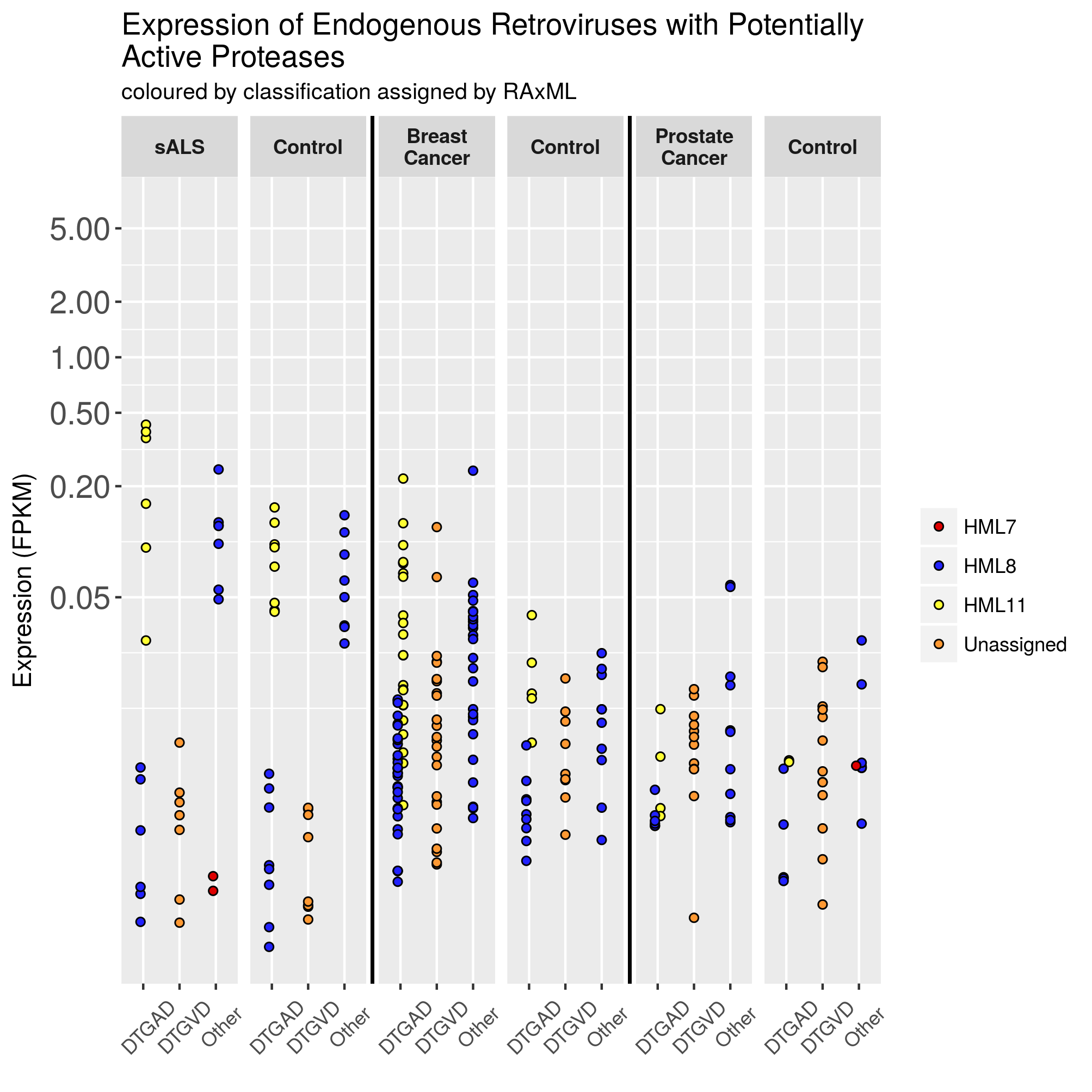

Supplement: FILE 1 in FASTA (fa) format — Aligned translated genomic protease BLAST results. The sequences found by BLAST for the HK2 protease in the human genome aligned and translated by MACSE. [file Data_Sheet_1.zip › Additional file 42.png]

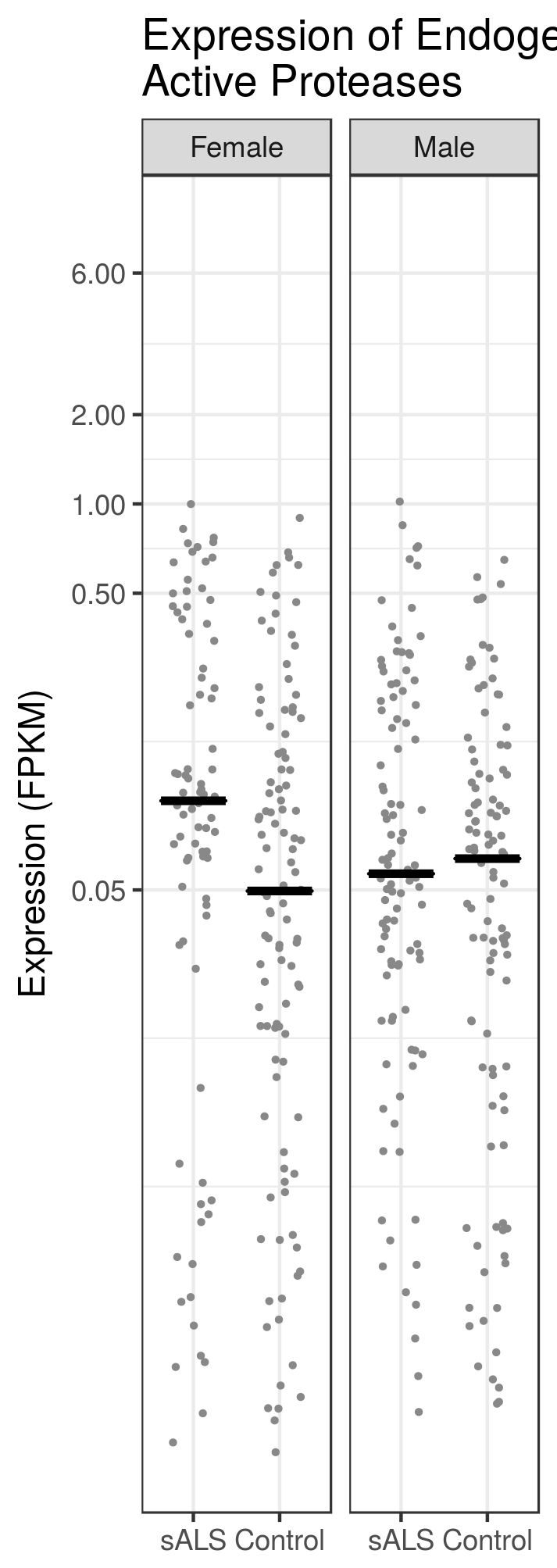

Supplement: FILE 1 in FASTA (fa) format — Aligned translated genomic protease BLAST results. The sequences found by BLAST for the HK2 protease in the human genome aligned and translated by MACSE. [file Data_Sheet_1.zip › Additional file 43.png]
